# Long-term trends in heavy metal contamination of marine sediments in the Arabian Gulf: A meta-analysis

**DOI:** 10.1007/s10661-025-14348-0

**Published:** 2025-07-08

**Authors:** S. Swetha, S. Veerasingam, S. Rajendran, Hassan Hassan, Muhammad Zia U. R. Rahman Hashmi, Hamood Alsaadi, Nelson Rangel-Buitrago, Fadhil N. Sadooni

**Affiliations:** 1 Environmental Science Center, Qatar University, P.O Box 2713, Doha, Qatar; 2 Programas de Física - Biología, Facultad de Ciencias Básicas, Universidad del Atlántico, Barranquilla, Colombia

**Keywords:** Heavy metals, Marine sediments, Contamination factor, Geoaccumulation index, Pollution load index, Ecological risk index

## Abstract

Heavy metal (HM) contamination in marine sediments is a critical environmental concern due to its potential ecological and human health impacts. This study provides a comprehensive assessment of the spatial and temporal evolution of HM contamination in Arabian Gulf sediments over a 33-year period (1991–2024). Contamination levels were evaluated using key sediment quality indices such as the contamination factor (CF), geoaccumulation index (I_g_ₑₒ), pollution load index (PLI), and ecological risk index (ERI) based on available meta-data. Spatial analysis revealed that marine sediments along the Iranian and Saudi Arabian coasts exhibited higher contamination levels compared to those of other Gulf countries. This elevated contamination is likely attributed to industrial discharges, oil spills and effluent releases from desalination plants. Arsenic (As), cadmium (Cd), copper (Cu), nickel (Ni), and lead (Pb) emerged as the primary toxic contributors to sediment pollution. Overall, HM contamination status in the region is predominantly low to moderate, with localized hotspots near industrialized coastal zones. The study highlights the need for scientifically informed waste management strategies to mitigate HM contamination, particularly in nearshore environments impacted by industrial activities. Furthermore, it recommends the implementation of continuous monitoring programs to track temporal trends and identify emerging contamination sources. These findings provide critical insights for environmental managers and policymakers in developing sustainable pollution mitigation frameworks and enhancing the resilience of marine ecosystems in the Arabian Gulf.

**Supplementary Information:**

The online version contains supplementary material available at 10.1007/s10661-025-14348-0.

## Introduction

The contamination of HMs in marine environments, particularly in the Arabian Gulf (hereafter referred to as the Gulf), is a critical area of research due to the region's unique characteristics. These include its semi-enclosed basin, shallow water column, elevated evaporation rates, limited water exchange, and substantial anthropogenic activity. Combined with the residual impacts of the Gulf Wars, these factors contribute to the region’s heightened vulnerability to contamination (Naser, 2013). The Gulf has experienced two major oil spills in recent decades: the first during the Iran–Iraq War in 1983, and the second during the 1991 Gulf War (Janadeleh & Jahangiri, 2016). Moreover, the Gulf is heavily influenced by anthropogenic sources of metal contamination, including emissions from oil and petrochemical industries, fishing industries, industrial cities, shipping, residential and commercial development, agriculture, mining, cement, and stainless-steel production, and wastewater treatment plants. While anthropogenic activities are the primary contributors, regional geology also plays a vital role in understanding the distribution and migration of HMs in marine sediments (Al-Taani et al., 2014; El-Sorogy et al., 2016; Veerasingam et al., 2014).

In certain areas of the Gulf, atmospheric dust deposition has been identified as the main source of lithogenic particles in surface marine waters (Yigiterhan et al., 2020). The environmental challenges posed by HMs are significant, even affecting indoor air quality in the region (Mahfouz et al., 2019). Between 1970 and 1990, various industries were established along the coastline and near small cities, a trend that persists and continues to contribute to metal contamination (Alharbi et al., 2017; Janadeleh et al., 2018; Karbassi & Bayati, 2005; Loughland et al., 2012). Although HMs naturally occur in marine ecosystems, they often enter aquatic systems through human activities (Aali et al., 2024; Nour & Nouh, 2020; Nour et al., 2022a). Their accumulation in marine organisms can exceed established safety thresholds, posing risks to both ecological health and human well-being.

Despite their biological necessity in trace amounts, HMs are considered persistent pollutants due to their chemical stability, low degradability, and tendency to bioaccumulate in protein-rich tissues through their cationic forms (Chakraborty et al., 2010; Ali & Chidambaram, 2021; Egbueri et al., 2022; Hassan et al., 2022; Aali et al., 2024). In coastal ecosystems, HMs predominantly accumulate in clays, oxides and sulfides (Ruilian et al., 2008), while their secondary accumulation in sediments is governed by redox conditions, biological activity, and local hydrography (Smrzka et al., 2019). Sediments thus serve as crucial indicators for assessing contamination levels and evaluating aquatic ecosystem health due to their ability to accumulate various pollutants. Studies have shown that more than 90% of HM contamination is associated with suspended particulate matter and sediments (Agah et al., 2016; Amin et al., 2009; Zheng et al., 2008). As key sinks for these metals, sediments also facilitate the remobilization of pollutants, allowing them to re-enter the water column and impact marine biota. Hence, HM levels in sediments are considered reliable indicators of toxicity in marine environments, as they exhibit more stable and consistent concentrations than those found in seawater (Allen, 1995; Soylak et al., 2002; Ruiź-Fernández et al., 2003; Cuong & Obbard, 2006; Alomary & Belhadj, 2007; Peer & Safahieh, 2011; Janadeleh & Kameli, 2017; Hasna et al., 2024). Investigating HM levels in sediments is essential for continuous pollution monitoring and identifying the types and sources of contamination (Al-Kahtany et al., 2023a, 2023b; Nour et al., 2022b).

However, evaluating contamination based solely on metal concentrations can lead to skewed interpretations, as it may overlook other contributing factors. Over the past three decades, various studies have assessed HM contamination status using indices such as the contamination factor (CF), pollution load index (PLI), geoaccumulation index (Igeo), and ecological risk index (ERI) (Abou Elezz et al., 2022; Agah et al., 2012; Aghadadashi et al., 2019; Al-Kahtany et al., 2023a, 2023b; Chakraborty et al., 2014; Mirzaei et al., 2020; Veerasingam et al., 2012, 2015; Venkatachalapathy et al., 2011). This study aims to (i) investigate the spatial and temporal distribution of metals in the marine sediments of the Gulf, (ii) analyze the variations in CF, PLI, I_geo_ and ERI in relation to metal contamination trends over time, and (iii) assess the impact of anthropogenic activities on HM accumulation in marine sediments. The outcomes of this investigation are expected to provide valuable insights for environmental professionals in diagnosing and mitigating HM contamination in marine sediments of the Gulf. Furthermore, it aims to inform stakeholders and broader scientific community about the extent and nature of HM contamination, while highlighting gaps in existing literature to support future research and sustainable coastal management.

## Geology and climate of the Arabian Gulf

The Gulf can be geologically classified into four main tectonic regions: i) the Precambrian shield region, primarily composed of metamorphic and igneous rocks, located in western Saudi Arabia; ii) the stable shelf region, encompassing eastern Saudi Arabia, the United Arab Emirates (UAE), Qatar, Bahrain, Kuwait and southwestern Iraq, which consists mainly of sedimentary deposits; (iii) the unstable shelf region, covering northern, central and eastern Iraq as well as southwestern Iran, which is tectonically more active; and (iv) the Tethyan Geosyncline region, comprising parts of the eugeosynclinal and miogeosynclinal belts, including the northeastern areas of Iraq, the Oman mountain belt, and the adjoining Zagros fold belt in Iran (Mukhopadhyay et al., 1996). Geographically, the Gulf is situated between the northwest and southeast of the Arabian Peninsula. Its width narrows at the Strait of Hormuz, which connects it to the Arabian Sea, a part of the Indian Ocean. The Gulf receives significant freshwater inflows from major river systems in the northern and northeastern parts of Iran, including Hilleh, Mand and Hendijan Rivers. Additionally, the Shatt Al-Arab River—formed by the confluence of the Tigris, Euphrates and Karun Rivers—flows into the Gulf from the northwest (Massoud et al., 1996).

**Fig. 1:**
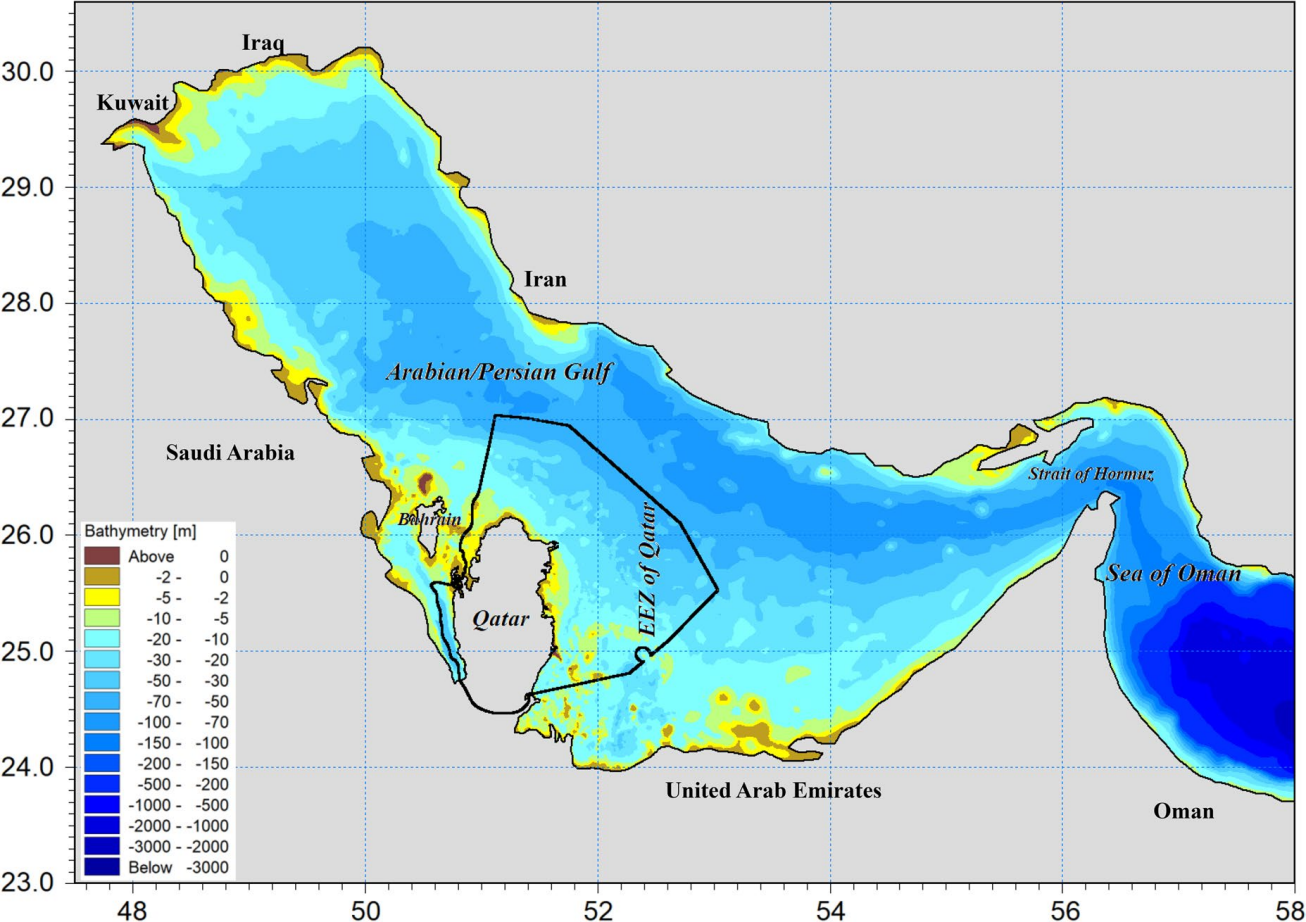
Bathymetry map of the Arabian Gulf

The Gulf features a shallow bathymetric profile, particularly along its northwestern and western coastlines, with an average depth of 50 m and a maximum depth of 90 m (Reynolds, 1993) (Fig. 1). The basin floor is asymmetrical, with its deepest axis situated closer to the Iranian coastline, transitioning from a shallow deltaic zone in the north to deeper waters in the south. The Gulf spans an area of approximately 251,000 km^2^ (96,912 square miles) and connects to the Gulf of Oman through the Strait of Hormuz to the east. Its western boundary is defined by the Shatt al-Arab delta, also known as"Arvand Rud"(meaning"Swift River"), where the Tigris and Euphrates converge. The southwestern shoreline of the Arabian Peninsula is predominantly linear, with the exception of the Qatar Peninsula, which disrupts the current and tidal patterns in the region. The Gulf stretches 989 km (615 miles) in length, with Iran occupying most of the northern coastline and Saudi Arabia dominating the southern shore.

The climate of the Gulf is arid and characterized by two distinct seasons: winter and summer. Winter (November-March): Daytime temperatures typically reach 26 °C, while nighttime lows drop to approximately 15°C. Summer (April–September): Temperatures often exceed 50 °C, with coastal cities frequently experiencing highs above 40 °C, accompanied by relative humidity levels approaching 90%. Colder temperatures are observed in elevated mountainous areas, where winter temperatures range between 10 °C and 14 °C during January and February. Annual precipitation is sparse, averaging between 140 and 200 mm. However, Oman receives significantly more rainfall, averaging around 350 mm annually. Most of the rainfall occurs during winter, driven by interaction of cold northern winds with weather systems originating from the Indian Ocean. In contrast, summer rainfall is minimal, especially in low-lying coastal regions, and in some years, no precipitation is recorded. The region is also prone to frequent sand and dust storms, primarily driven by the Al–Shamal wind. These events can contribute significantly to the deposition of HM concentrations in the Gulf’s seawater.

## Regional distribution of sediments

The distribution of sediments in the Gulf is primarily influenced by several interrelated factors, including the region’s prevailing arid climate, variations in wave energy, the orientation of the coastline relative to the northwesterly Shamal winds, and the presence or absence of offshore barriers. The southwestern coast is mainly characterized by carbonate deposits interspersed with eolian clastics and is affected only by Alpine orogeny. In contrast, the Iranian coastline is rocky and linear, featuring a combination of terrigenous sedimentation and carbonate deposition. Finer sediment fractions are predominantly found in the northern regions of the Gulf, indicating significant fluvial input and deposition. Along the southern coast and adjacent offshore areas, sediments are primarily composed of bioclastic and oolitic sands, indicative of high-energy environments. Bioclastic sediments are observed at depths of up to 20 m, indicating that the storm wave base extends to at least this depth (Purser & Seibold, 1973). Beyond 20 m, the Gulf experiences low- to moderate-energy conditions, where fine-grained argillaceous and micritic sands dominate sediment accumulation (Houbolt, 1957; Purser & Seibold, 1973). The euphotic zone–the depth to which sufficient sunlight penetrates to support photosynthesis–varies throughout the Gulf. In the southern coastal areas, the presence of algal growth indicates that the euphotic zone extends to about 20 m. In contrast, in clearer waters along the central axis of the Gulf, it can reach depths of up to 30 m (Purser & Seibold, 1973).

## Data and methods

### Data used

**Fig. 2:**
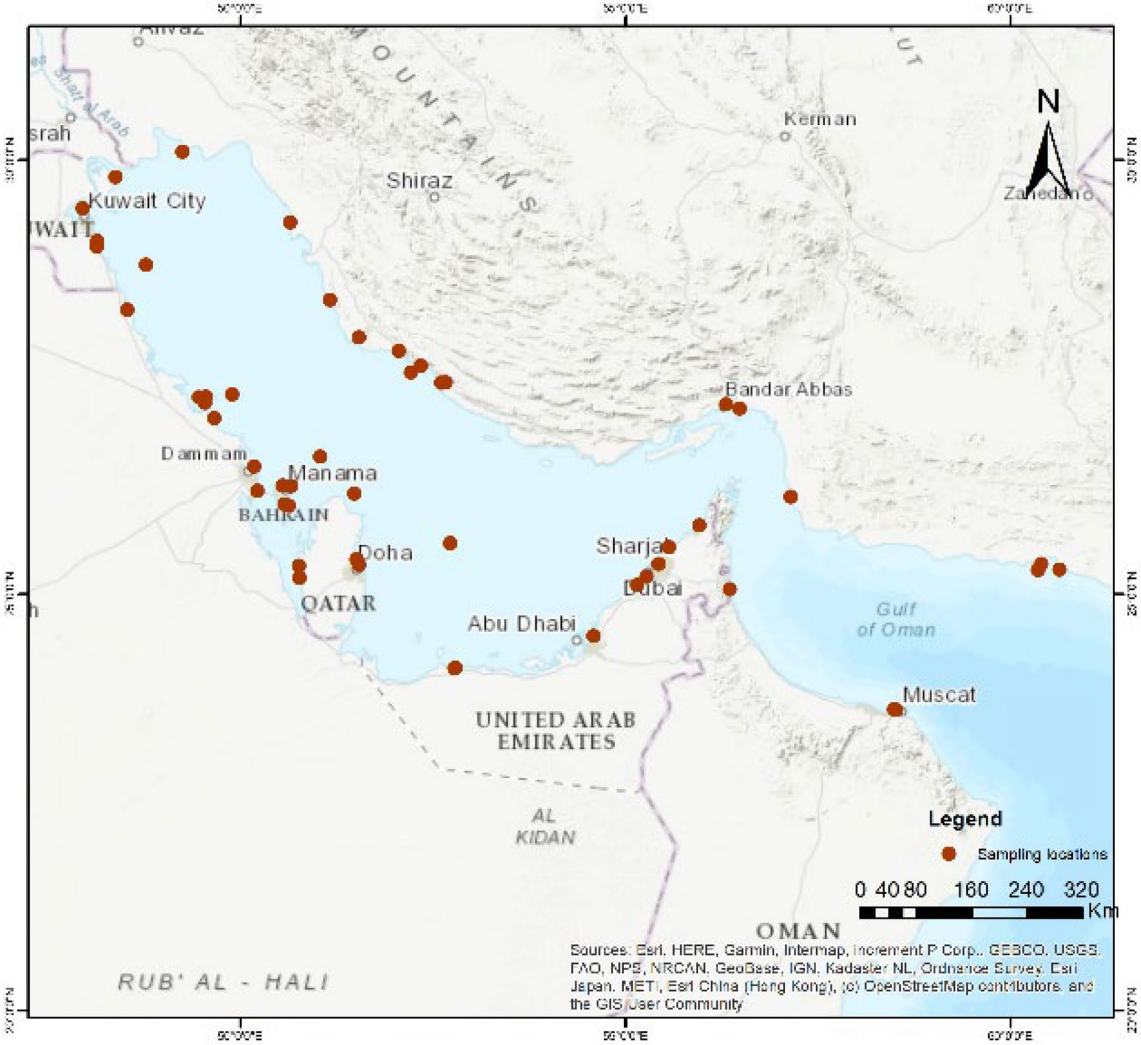
Spatial overview of representative sampling locations used in the metadata analysis

This study utilized 56 datasets on HM contamination collected from 47 peer-reviewed research papers published between 1991 and 2024. For the spatial analysis, ArcGIS 10.8 software was used to provide a comprehensive visualization of HM distribution and contamination trends over a period of 33 years. Among the 47 peer-reviewed research papers used, 6 were from Qatar, 3 from Bahrain, 9 from Saudi Arabia, 5 from Kuwait, 2 from Iraq, 15 from Iran, 2 from Oman, and 5 from the UAE (Fig. 2).

### Literature search strategy and study selection criteria

A comprehensive systematic literature search was conducted to identify peer-reviewed studies investigating HM contamination in marine sediments. Studies published between 1991 and 2024 were included if they met the following criteria: (i) peer-reviewed status, (ii) reported either mean concentration values for the entire study area or provided data for individual sampling locations, (iii) included precise spatial documentation, either through geographic coordinates or detailed location maps, and (iv) employed standardized analytical methods such as ICP-MS or AAS to ensure data reliability and comparability. Studies were excluded if they (i) were not peer-reviewed, (ii) focused on regions outside the Arabian Gulf, (iii) lacked adequate spatial information for sampling locations, or (iv) did not report HM concentration data specific to marine sediments.

### Risk assessment of HMs in marine sediments

In this study, both simple and integrated indices were applied to evaluate the levels of HM contamination. These indices provide a comprehensive understanding of pollution by quantifying the degree of contamination and assessing the potential ecological risks posed by HMs in marine sediments.

### Contamination factor (CF)

The contamination factor (CF) is a simple index used to evaluate the pollution status of individual HM in sediments. It is calculated using the following equation:

1 $$CF=\frac{Metal\;content\;in\;the\;sediment}{Background\;value\;of\;the\;metal}$$

Here, the ‘‘Background value of the metal’’ refers to the concentration (ppm) of HMs in unpolluted sediments. Due to the lack of specific background values for Gulf sediments, we adopted the upper continental crust (UCC) values proposed by Taylor and McLennan (1995). The CF values were classified according to Hakanson (1980) as follows: CF < 1: Low contamination, 1 ≤ CF < 3: Moderate contamination, 3 ≤ CF < 6: Considerable contamination, and CF ≥ 6: High contamination.

### *Geoaccumulation Index (I*_*geo*_*)*

The geoaccumulation index (I_geo_) quantifies the intensity of contamination for each HM (Muller, 1969). It compares the current concentration of a metal in the sediment with its background value, using the following equation:

2 $${\text{I}}_{\text{geo}}={\text{log}}_{2}\frac{\text{Cn}}{1.5\text{Bn}}$$

where C_n_ is the current concentration of the HM in the sediment, and B_n_ is the geochemical background concentration (as used in the CF assessment). The factor of 1.5 is used to account for natural lithological variations in the metal concentrations. The I_geo_ values are interpreted as follows:

I_geo_ < 1: Uncontaminated to minimally contaminated.
I_geo_ = 1–2: Moderately contaminated.
I_geo_ = 2–3: Moderately to heavily contaminated.
I_geo_ = 3–4: Heavily contaminated.
I_geo_ = 4–5: Heavily to extremely contaminated.
I_geo_ > 5: Extremely contaminated.

### Pollution load index (PLI)

The pollution load index (PLI) assesses the overall contamination status of sediment samples based on the combined CF values of selected HMs. It is expressed as:

3 $$PLI=\sqrt[ n]{CF1\times CF2 \times CF3 \times \dots \times CFn}$$

where n is the number of metals assessed. The interpretation of the PLI values, based on Tomlinson et al. (1980), is as follows:

PLI < 1: Good sediment quality.
PLI = 1: Baseline level of pollution.
PLI > 1: Progressive deterioration of sediment quality.

### Ecological risk index (ERI)

The ecological risk index (ERI) is used to evaluate the potential ecological risks posed by HMs. It is calculated as the sum of the products of the CF and the corresponding toxicity response factor (TR) for each metal:

4 $$ERI=\sum \left(CF\times TR\right)$$

The TR values used for the selected HMs are: Cd (30), As (10), Cr (2), Co (5), Cu (5), Fe (1), Mn (1), Ni (5), Pb (5), V (2), and Zn (1). The classification of ERI values is presented in Table S1.

## Results and discussion

### Spatial and temporal distributions of HMs in marine sediments in the Gulf

The sedimentary composition of the Gulf results from the combined interactions of marine, riverine and aeolian processes that deposit sedimentary materials in the region. The average concentrations (ppm) of HMs in marine sediments reported in studies conducted over the last 33 years (1991–2024) are summarized in Table 1. The highest concentrations (in ppm) of HMs were reported in the following order: Cr (4090) > Zn (1690) > Mn (1354) > Pb (1347.44) > Cu (769) > V (759.15) > Ni (497.46) > As (283) > Co (30.14) > Cd (19.45). These values indicate significant spatial and temporal variability across the region. The highest concentrations of HMs, such as As (283), Cr (4090), Cu (769), Mn (1354), V (759.15), Cd (19.4), Pb (1347.44), and Al (4500) were frequently reported in sediments from Saudi Arabia and Iran. These elevated values were observed across consecutive years, with As, Cr, Cu, and Mn detected in Saudi Arabian sediments in 2016; V in 2018; Cd and Pb in Iranian sediments in 2019; and Al in 2021. The highest Fe (112100 ppm) concentrations and Zn (1690 ppm) concentrations were reported in sediments from Qatar (2003), while Ni (497.46 ppm) and Co (30.14 ppm) peaked in the United Arab Emirates (2017) and Iraq (2007), respectively. In contrast, the lowest concentrations of Zn (1.28 ppm), Cd (0.02 ppm), Cu (1.2 ppm) and Mn (11.7 ppm) were recorded in Kuwait (1993). Recent studies have highlighted elevated concentrations of various HMs in regions impacted by industrial activities. The key contributors to HM pollution in the Gulf include (i) industrial zones – proximity to refineries and petrochemical plants; (ii) ports – intense maritime activity leading to localized contamination; and brine disposal – discharge of effluents contributing significant HM loads.

**Table 1:** Distribution of mean value concentrations (ppm) of HMs in the Gulf

Country	Year of study	As	Al	Cd	Cr	Co	Cu	Fe	Mn	Ni	Pb	V	Zn	Reference
Kuwait, Qaruh Islands	1991	32.6		0.77	2.85	0.91	1.2	2792	11.7	15	1.03	13.2	1.28	Fowler et al. (1993)
Saudi Arabia	1993	1.08		4.14	15.42		9.06		35.78	32.42	1.54	7.96	8.86	Al-Arfaj and Alam (1993)
Kuwait	1994			0.02	55.5					30.8	1.95	17.3		Al-Muzaini and Jacob (1996)
Saudi Arabia	1998				32		10	5300	133	42		21	26	Basaham and El-Sayed (1998)
Arabian Gulf	1998		0.87		38		9	6900	165	30		19	22	Basaham and El-Sayed (1998)
Abudhabi	1998			5.9	10.7	10.7	3.5		112.7	2.4	23.8		10.9	Shriadah (1998)
Dubai	1998			5.3	10.8	10.4	3.8		116.9	27.2	28.1		42	Shriadah (1998)
Sharjah	1998			5.7	18.8	12.8	29.3		110.1	20.6	43.6		300.3	Shriadah (1998)
Umm al-Quwain	1998			5.8	9.1	10.5	18.2		82.4	17.1	25.4		18.7	Shriadah (1998)
Ras al-khaima	1998			5	8.6	9.9	16.9		61.1	17.8	26.3		13.6	Shriadah (1998)
Qatar	2001	4.375	7176.8	0.07	27.08	1.32	4.37	3283	70.14	11.23	2.42	17.2		De Mora et al. (2004)
UAE	2001	3.06666	10,219	0.071666	107.4	9.64	2.343333	8665.666	188.8	197.2333	2.28	16.05		De Mora et al. (2004)
Qatar	2003				2.9	9.2	114	112,100	792	42.7	750		1690	Ahmed and Abdel-Moati (2003)
Baharin	2003			0.25115	42.93		36.17		43.47	13.21	20.35		38.35	Juma (2005)
Baharin	2004	5	4326.25	0.08225	17.79	0.898	14.135	2495.5	44	9.02	28.84075	12.2775	21.41	De Mora et al. (2004)
Oman	2004	2.505	7072	0.16	70.96	3.071833	2.7715	5429.833	122.3833	37.53833	1.110167	20.02		De Mora et al. (2004)
Iran	2004	7		0.2		14	20		357	111	7		37	Agah et al. (2012)
Iran	2005			2.894						64.897	90.479	52.003		Pourang et al. (2005)
Iraq	2006					30.14	14.04	7147.1	507.01	44.98			43.04	Al-Saad et al., 2007
Iranian coasts of the Oman Sea	2008						38.28			26.9	50.37			Peer and Safahieh (2011)
UAE	2008		7000				5.71	3600	70	8.89		15.44	21.67	Abd El-Gawad et al. (2008)
Iran	2010		30,572.8				15.432	24,319.6	321.3	56.04	6.524	31.52	48.16	Abdollahi et al. (2013)
Iran	2012	5.16	24,510.6	0.11	58.48	6.9	17.18	17,284.99	264.99	57.77	8.07	39.9	55.53	Aghadadashi et al. (2019)
Iranian coasts of the Oman Sea	2012						16.45			14.68	13.56			Peer and Safahieh (2011)
Qatar	2012						2.7			2.26		6.21	6.49	Al-Naimi et al., 2015
Iran	2013	7.4	4.9	0.12	322		30.4	27,100	596	63.9	240		67	Hamzeh et al. (2013)
Oman	2014			1.8			13.6				10.5	42		Al-Husaini et al. (2014)
Kuwait	2014	4.734		0.268	175.61		34.28276			138.5103	14.6		89.45517	Lyons et al. (2015)
Iraq	2014					13.67	28.5			95.5	12		56	Al-Jaberi & Al-Dabbas, 2014
Iraq	2014	29.0671		4.65							54.6752			Abdulnabi et al., 2019
Hormuz Strait	2015				40.03		30.12	18,388		34.05	7.73			Janadeleh et al. (2018)
UAE	2015	2.8535		0.1		4.1	3.8			30.37321	1.9		8.2	Al-Rashdi et al. (2015)
Saudi Arabia	2016	1.65	2041	0.24	50.88	4.48	180.06	7681.9	116.06	75.94	5.22	250.24	53.53	Alharbi et al., (2017)
Iran & Gulf of oman	2016		19,000		210	8	15	25,000		32	26	64	30	Irandoost et al. (2021)
Iran (Oman sea)	2016	13.22			185.01	8.91	14.16			40.51	12.87	47.21	37.86	Agah et al. (2016)
Saudi Arabia	2016	283	30,015		4090		769	39,292	1354	3.07	9.35			Alshahri (2017)
Persian Gulf	2017			0.53	18.89		2.03	59.84		20.13	9.51		47.99	Mirzaei et al. (2020)
Iran	2017	6.2	45,000	2.29	92.3	13	14.1	31,000	422	58	9.2	45.5	39.6	Agah, 2021
Kuwait	2017			1.44	36.48		16.48	9743		42.46	5.22	26.38	36.62	Al-Qattan & Al-Sarawi (2017)
UAE	2017	15.14		5.02	156.565	24.4643	9.00434	21,469.9	254.847	497.46	11.6226	19.4	17.15522	El-Tokhi et al. (2017)
Qatar	2018	1.43		0.26	12.2	0.75	4.72		53.17	6.22	2.08	7.73	8.32	Castillo et al. (2024)
Qatar	2018	5.81		0.034	9.94	1.68	5.57	3184.95	68.86	10.15	1.25		7.44	Alshuiael et al., 2022
Saudi Arabia	2018	30.61	1887.07	2.13	63.79	4.01	297.29	8474.21	111.57	77.07	5.25	603	48.26	El-sorogy et al. (2018)
Saudi Arabia	2018	23.745	1991.7	1.6715	50.65	3.94	209.8	7946.9	122.5	81.05	4.395	759.15	28.35	Al-Kahtany et al. (2018)
Kuwait	2019				67.9	8.31	13.7			61.1	5.99		41.6	Alshemmari & Talebi (2019)
Kingdom of Bahrain	2019	5.173		0.2673	41.53		55.42			24.896	29.516		68.1142	Bersuder et al. (2020)
Iran	2019			19.45	34.2		465			7.09	1347.44		427.16	Arfaeinia et al. (2019)
Iran	2019			1.73	5.3		79.9			2.55	64.09		97.45	Arfaeinia et al. (2019)
Iran	2019			5.06	11.09		116.84			7.45	164.89		247.88	Arfaeinia et al. (2019)
Iran	2019			0.83	1.94		6.34			1.18	3.18		27.43	Arfaeinia et al. (2019)
Iran	2020						11.59		121.47	11.51	5.3			Allami et al. (2020)
Iran	2020						12.56		131.59	10.81	4.88			Allami et al. (2020)
Iran	2021	3.9			72.9	9.4	17.5	14,000		59.8	12.1	43.4	35.7	Aali et al. (2024)
Saudi Arabia	2021	2.47			7.86	1.43	4.14	4808.33		13	3.5	6.67	6.89	El-Sorogy & Al-Kahtany (2022)
Iran (MPAs)	2022	6.71	24,700		88.67	7.5	12.5	10,200	327.3	54.83	14.65	37.5	24.4	Ghaemi et al. (2023)
Qatar	2022	2.56	6500	0.52	13.3	0.99	4.82	1972	28.2	11.4		8.46	6.98	Hasna et al. (2024)
	2022	2.45	16,970		33.73	1.89	9.23	6876	151	18.3	1.96	25.8	49.4	Hasna et al. (2024)
Western Arabian gulf coastal area	2022			0.168	6.844		2.294	1460	34.3	3.932	0.919		6.939	Amin & Almahasheer (2022)
Saudi Arabia	2023	2.47			7.86	1.43	4.14	4808		13	3.5	6.67	6.89	Al-Kahtany & El-Sorogy, 2023
Saudi Arabia	2023	2.38			8.68		2.44			11.76	2.57		6.18	Alzahrani et al. (2023)

### Risk assessments of HMs in marine sediments in the Gulf

To accurately estimate anthropogenic inputs to marine sediments, background concentrations of HMs were determined using upper continental crust (UCC) values (Taylor & McLennan, 1995). These background concentrations (in ppm) for the selected HMs are as follows: Cd (0.098), Cr (35), Cu (25), Mn (600), Ni (20), Pb (20), V (60), Co (10), As (1.5) and Zn (71).

**Fig. 3:**
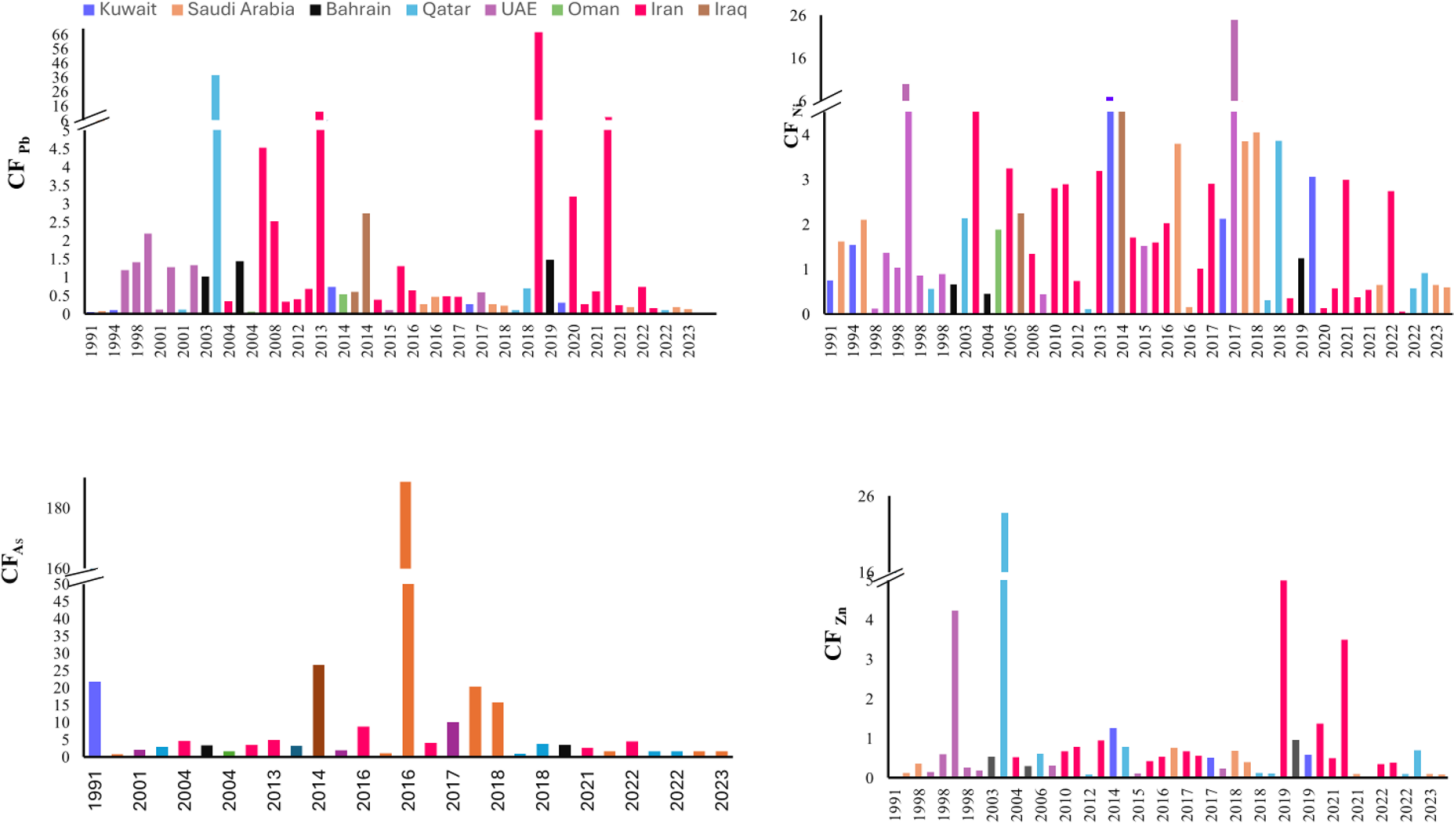
Temporal changes in the contamination factors of HMs in the Gulf sediments

Figure 3 illustrates the temporal variations in the CF values for the selected HMs in the Gulf, with detailed numerical data available in Table S2. Exceptionally high CF values were reported for As (188.67), Cr (116.86), Cu (30.76), and Mn (2.26) in Saudi Arabian sediments, particularly near brine disposal sites associated with desalination plants. Elevated vanadium concentrations in sediments from Ras Abu Ali Island are linked to oil spill incidents in the region (Alharbi et al., 2017). The highest CF value for Ni (24.87) was recorded along the UAE coast, attributed to chronic oil pollution. Elevated CF values for Cd (198.47) and Pb (67.37) were observed along the eastern coast of the Gulf, particularly at Asalouyeh Port (Iran), due to substantial industrial discharges. An extremely high CF value for Al (5597.012) was recorded at Posm and Ramin in Iran, primarily due to terrestrial inputs of aluminosilicate minerals. In Qatar, CF values for Fe (23.80) and Zn (32028.57) were found to be abnormally high. The elevated Fe level is attributed to the loading and unloading of iron ore at the Iron & Steel Complex, while the high Zn level is likely due to its strong scavenging affinity for Fe–Mn oxyhydroxides, which are prevalent in the sediment matrix of the area. Excessive Co concentrations (CF = 3.01) were observed along Khor Al-Zubair (Iraq), possibly driven by pollution associated with power stations, sewage effluent, industrial and port facilities, agricultural runoff, coastal construction, and oil transport activities.

Meta-analysis revealed that Pb contamination is generally low across the Arabian Gulf, except in the locations. In contrast, Cd concentrations are significantly high along major coastlines, with peak values recorded along the eastern Gulf coast.

### Pollution load levels of HMs in sediments in the Gulf

**Fig. 4:**
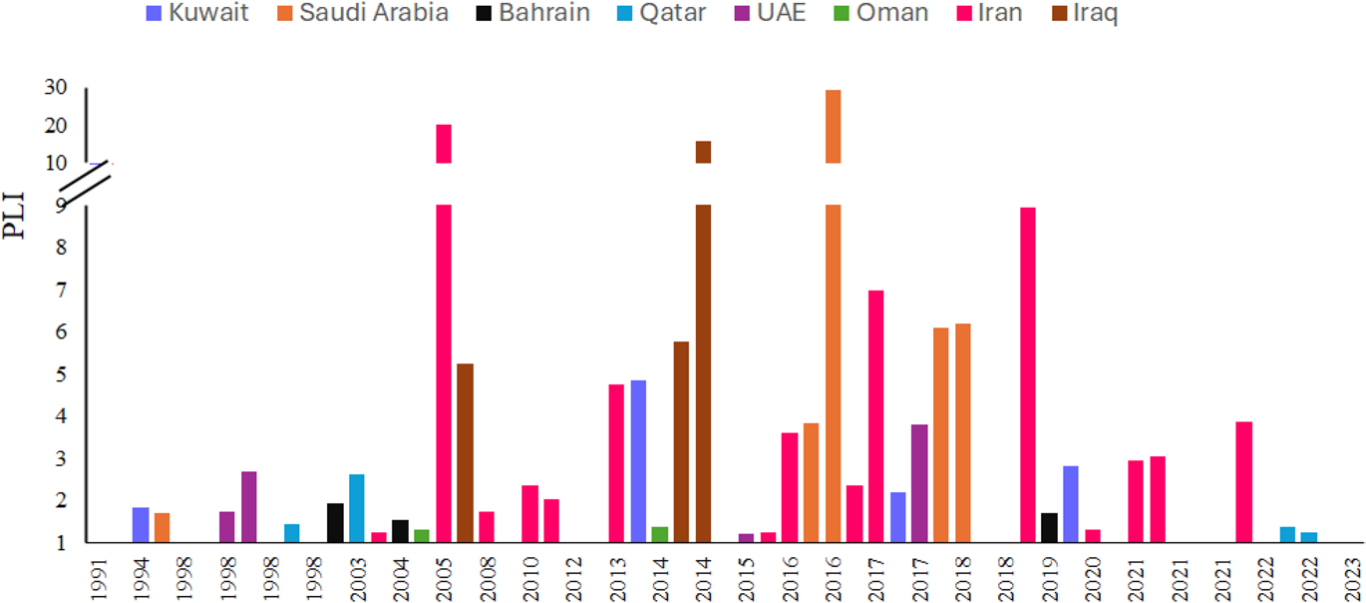
Temporal changes in the PLI values of sediments across Gulf

The PLI was calculated to assess overall contamination trends in marine sediments across the Gulf (Fig. 4 and Table S2). The results indicate that sediments along the western coastline exhibited higher contamination levels compared to those along the eastern coastline. Over the 33 years, most sediment samples showed PLI values below 3, indicating progressive deterioration in sediment quality based on the adopted classification scales. However, our study suggests that variation in these values may also depend on the number and type of HMs selected for analysis. Moreover, significant variability in HM loading can be attributed to fluvial inputs from Iraq (Al-Jaberi & Al-Dabbas, 2014), sediment transport by long-shore currents in the Gulf (Sheppard et al., 2010), as well as dust deposition from Shamal winds (Yigiterhan et al., 2020) and other anthropogenic disturbances affecting sediment quality across the Gulf.

The calculated PLI values across the Gulf generally showed minimal variation, except in Saudi Arabia (PLI = 29.43), Iran (PLI = 20.16) and Iraq (15.76), where exceptionally high values were recorded. These elevated levels are likely due to contamination near desalinization plants and industrial zones. The PLI value for Kuwait’s marine sediments (4.84) is notably higher than that of nearby coastal areas such as Bahrain and Qatar, possibly due to sewage outfalls near the sampling sites.

Industrial waste discharges, especially near port areas, along with effluents from desalinization plants are considered major contributors to elevated metal loads along both eastern and western Gulf coastlines. Additionally, particle size plays a significant role in influencing contamination levels: finer sediments generally retain more HMs due to their higher surface area and adsorption capacity. Interestingly, higher contamination levels have also been observed in coarser sediments in some locations, likely due to localized anthropogenic sources, limited sediment transport, and longer retention times. Recent studies show a notable decline in PLI values, with some proximal regions recording values as low as 0.25, indicating improved sediment quality. A study conducted in the western Gulf in 2022 reported the lowest PLI value (0.15) in the past three decades, further confirming this positive trend. Overall, PLI values across the Gulf countries are relatively similar, with the exception of Saudi Arabia, which highlights the need for more effective pollution control and monitoring measures in that region.

### Geo-accumulation patterns of HMs in the marine sediments of the Gulf

**Fig. 5:**
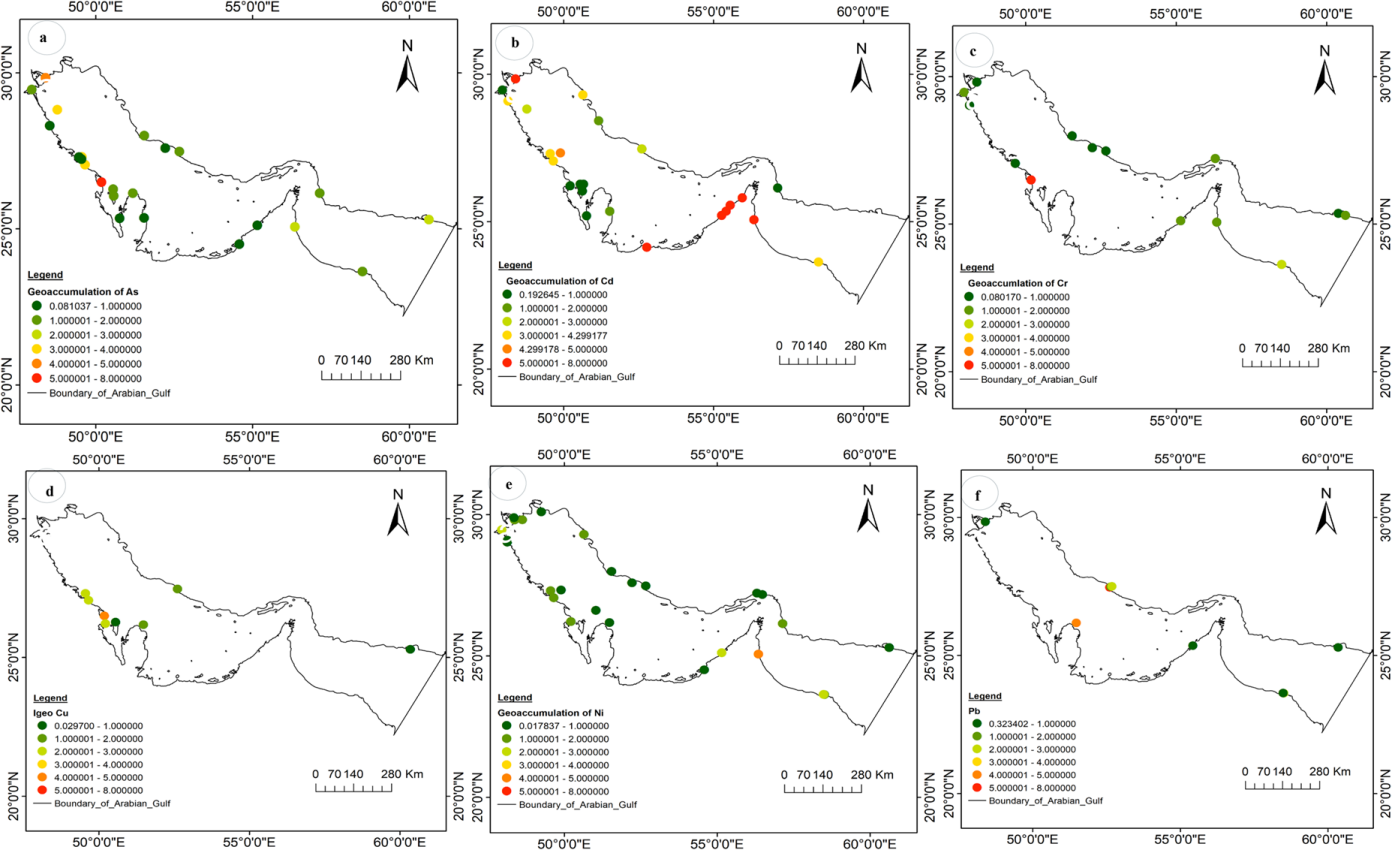
Geo-accumulation Index of **a**) As, **b**) Cd, **c**) Cr, **d**) Cu, **e**) Ni, and **f**) Pb in marine sediments across the Gulf under the period 1991 to 2024

The geoaccumulation index (I_geo_) for selected HMs in the marine sediments of the Gulf over the past 30 years is illustrated in Fig. 5. For this analysis, six HMs (As, Cd, Cr, Cu, Ni, and Pb) with I_geo_ values greater than zero were selected, highlighting their relevance for discussion. The I_geo_ trends of HMs over 33 years reveal contamination levels ranging from minimal to very severe. The I_geo_ value for As reached a maximum of 6.97 in Saudi Arabia, followed by Iraq with an I_geo_ of 4.15 (Fig. 5a). In contrast, Cd showed particularly high contamination along the western coastline, predominantly within the UAE jurisdiction, where I_geo_ values exceeded 5, indicating extreme levels of contamination. (Fig. 5b).

Severe I_geo_ values for Cr (6.28) (Fig. 5c), As (6.97) and Cu (4.35) were observed in the same region of Saudi Arabia, where anthropogenic activities are most concentrated, as discussed in relation to the CF and PLI. Moreover, proximal sites in this region were found to be transitioning from moderate to severe contamination, particularly for Cu (Fig. 5d) and As, emphasizing the need for continuous sediment monitoring. The reported I_g_ₑₒ values for Pb range from minimal to very severe contamination, with the highest value (5.48) recorded in Iran, followed by 4.64 in the Exclusive Economic Zone (EEZ) of Qatar (Fig. 5f). For Ni, most I_g_ₑₒ values indicate minimal to moderate contamination, with the highest value of 4.05 reported in the UAE (Fig. 5e). Overall, the I_g_ₑₒ values for HMs in Gulf marine sediments suggest that Cd, Ni, Pb, As and Cu are the primary toxic contributors to pollution in the region, significantly elevating the overall contamination status of marine sediments.

### Ecological risks of HMs in marine sediments of the Gulf

**Fig. 6:**
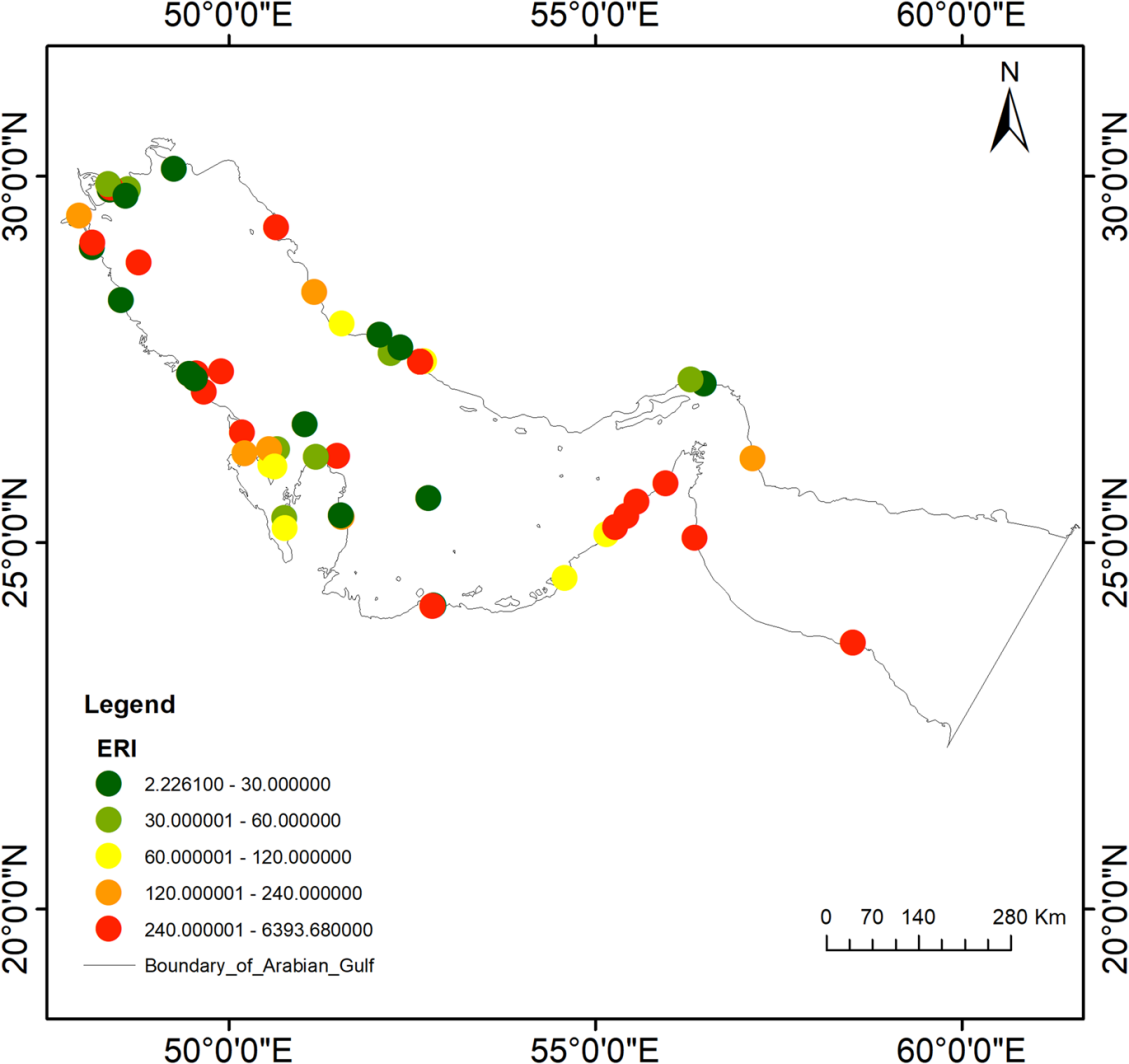
Ecological Risk Index of HMs in Sediments from Coastal Regions of the Gulf

Figure 6 and Table S2 present the total ecological risk index (ERI) values for the metals As, Cd, Cr, Co, Cu, Mn, Ni, Pb, V, and Zn in the marine sediments of the Gulf. The ERIs were calculated using the toxic response factors assigned to each metal. Over the past 33 years, the highest ERI value of 6393.68 was recorded in Iran in 2019. The frequency of elevated ERIs is notably higher along the western coastline of the Gulf compared to the eastern side. A long-term meta-data analysis revealed that 16 high-risk zones for HMs are distributed throughout the Gulf’s sediments. This study suggests that the wide variation in ERIs compared to other indices may be attributed to differences in toxic response factors, as well as the number and distribution of sites included for each metal and region. Based on the available data, the countries in the Gulf with the highest ERIs are ranked as follows: Iran (6393.68) > Saudi Arabia (2271.54) > Iraq (1944.43) > UAE (1819.67) > Oman (557.76) > Kuwait (458.39) > Qatar (250.86) > Bahrain (144.33). Notably, the UAE recorded five critical risk zones in 1999 near industrial areas. However, recent studies have shown significantly lower ERI values in the UAE and other regions, indicating a marked improvement in sediment quality.

### Environmental and methodological factors influencing HM variability

The findings indicate that the study-specific latent variables contribute significantly to the heterogeneity observed in HM concentrations over extended temporal scales (Table S3). This variability is largely attributed to methodological disparities in sampling times and protocols, sediment characterization techniques, and analytical procedures used across different investigations. Most studies focused on surface sediments rather than bottom sediments, with considerable variation in sampling depth protocols. Some investigations targeted only the top 0–5 cm surficial layers, while others included deeper sediment horizons or employed composite depth-integrated sampling approaches. Grain size heterogeneity also emerged as a major source of variability. Many studies analysed only fine-grained fractions (< 63 µm), which have a greater capacity to retain HMs due to their larger surface area and higher number of adsorption sites. In contrast, some studies examined bulk sediments or coarser fractions, potentially leading to a systematic underestimation of HM concentrations. The lack of mineralogical data across the reviewed studies is likely due to the dominance of industrial discharge sources in the study areas. When HMs originate from direct anthropogenic sources, their distribution is typically governed more by physical sediment characteristics such as grain size rather than indigenous mineralogical composition.

### Future research perspectives

The long-term assessment of HM contamination in Arabian Gulf sediments provides a crucial foundation for understanding pollution trends, identifying high-risk zones, and formulating effective mitigation strategies. Over the past three decades, industrial expansion, urbanization, and anthropogenic inputs have significantly altered sediment quality, with certain regions experiencing severe contamination. However, recent studies indicate a promising decline in pollution levels in some areas, suggesting that environmental regulations and improved waste management practices may be yielding positive results.

Despite this progress, several challenges remain. The variability in contamination factors highlights the need for standardized monitoring protocols across the Gulf region. Differences in analytical methods, sampling locations, and metal selection criteria have influenced reported pollution indices, emphasizing the importance of a harmonized approach to future research. Moreover, understanding the complex interactions between natural and anthropogenic processes, including sediment transport, hydrodynamic forces, and climate change-induced shifts in deposition patterns is essential for gaining comprehensive picture of contamination dynamics.

The integration of advanced geospatial tools, artificial intelligence, and predictive modeling holds the potential to revolutionize how marine pollution is assessed and managed. Techniques such as remote sensing, machine learning, and in situ monitoring networks can enhance real-time detection capabilities and improve forecasting, supporting more informed decision-making by policymakers and researchers. Moreover, interdisciplinary collaboration among marine scientists, environmental agencies, and local governments is essential for developing effective intervention strategies that promote the sustainability of marine ecosystems in the Arabian Gulf.

As the region continues to develop, balancing economic growth with environmental stewardship is critical. By implementing proactive measures, strengthening international cooperation, and fostering innovation in pollution monitoring and control, the Arabian Gulf can move toward a cleaner, healthier marine environment for future generations.

## Conclusions

This meta-analysis of HM contamination in Gulf sediments over three decades (1991 to 2024) provides crucial insights into the spatial and temporal distribution, pollution trends, and ecological risks associated with anthropogenic and natural influences. Iran and Saudi Arabia show the highest levels of HM pollution, primarily driven by industrial discharges, desalination effluents, and other anthropogenic sources. While certain sites show signs of improved sediment quality, persistently elevated concentrations of Cd, Cr, Pb, and As continue to pose significant ecological risks. The accumulation of HMs is further influenced by fine sediment composition and local geochemical conditions.

Despite these advances, the study highlights the pressing need for harmonized sampling and analytical methodologies across the Gulf to ensure data comparability and accuracy. Environmental factors such as sediment grain size and hydrodynamic transport, alongside methodological inconsistencies, contribute to the observed variability in reported HM concentrations and pollution indices. This study emphasizes the need for sustained and expanded monitoring efforts, the implementation of robust mitigation strategies, and the adoption of sustainable development practices to reduce HM loading into the Gulf’s marine environment. By documenting long-term trends and identifying critical hotspots, this study provides a foundational reference for policymakers, researchers, and environmental managers aiming to effectively address HM pollution in Gulf sediments.

This study recommends the integration of geospatial analytics, artificial intelligence-based modeling, and real-time monitoring tools to enhance the surveillance and forecasting of HM pollution in marine sediments. Regional cooperation, robust environmental governance, and interdisciplinary research will be pivotal in addressing ongoing challenges and securing a sustainable and resilient marine ecosystem in the Arabian Gulf.

## Supplementary Information

Below is the link to the electronic supplementary material.

Supplementary file1 (DOCX 42 KB)

Supplementary file2 (XLSX 50 KB)

## Data Availability

No datasets were generated or analysed during the current study.

## Declarations

All authors have read, understood, and complied as applicable with the statement on “Ethical responsibilities of Authors” as found in the “Instructions for Authors.” Ethics approval and consent to participate Not applicable.

## References

[CR1] Aali, A., Ghiyasi, S., Agah, H., Khoramnezhadian, S., & Saleh, A. (2024). Assessment of heavy metal content and ecological risk in offshore surface sediments of the Northern Persian Gulf: Implications for environmental management. *Regional Studies in Marine Science,**69*, 103317. 10.1016/j.rsma.2023.1033

[CR2] Abd El-Gawad, E., Lotfy, M. M., & El-Sammak, A. (2008). Assessing the environmental impacts of the Ruwais Industrial Complex (RIC), the offshore area, United Arab Emirates. *Australian Journal of Basic and Applied Sciences,**2*(4), 909–919.

[CR3] Abdollahi, S., Raoufi, Z., Faghiri, I., Savari, A., Nikpour, Y., & Mansouri, A. (2013). Contamination levels and spatial distributions of heavy metals and PAHs in surface sediment of Imam Khomeini Port, Persian Gulf, Iran. *Marine Pollution Bulletin,**71*(1–2), 336–345. 10.1016/j.marpolbul.2013.01.025 23523119 10.1016/j.marpolbul.2013.01.025

[CR4] Abdulnabi, Z. A., Altememi, M. K., Hassan, W. F., Al-Khuzaie, D. K. K., & Saleh, S. M. (2019). Assessing of some toxic heavy metals levels and using geo-accumulation index in sediment of Shatt Al-Arab and the Iraqi Marine region. *Baghdad Science Journal,**16*(2), 323–331. 10.21123/bsj.2019.16.2.0323

[CR5] Abou Elezz, A., Castillo, A., Hassan, H. M., Alsaadi, H. A., & Vethamony, P. (2022). Distribution and environmental geochemical indices of mercury in tar-contaminated beaches along the coast of Qatar. *Marine Pollution Bulletin,**175*, 113349. 10.1016/j.marpolbul.2022.113349 35092929 10.1016/j.marpolbul.2022.113349

[CR6] Agah, H. (2021). Ecological risk assessment of heavy metals in sediment, fish, and human hair from Chabahar Bay, Makoran, Iran. *Marine Pollution Bulletin,**169*, 112345. 10.1016/j.marpolbul.2021.112345 34238565 10.1016/j.marpolbul.2021.112345

[CR7] Agah, H., Hashtroudi, M. S., & Baeyens, W. (2012). Trace metals and major elements in sediments of the northern Persian Gulf. *Journal of the Persian Gulf,**3*(7), 45–58.

[CR8] Agah, H., Bastami, K. D., & Fumani, N. S. (2016). Ecological risk, source, and preliminary assessment of metals in the surface sediments of Chabahar Bay, Oman Sea. *Marine Pollution Bulletin,**107*(1), 383–388. 10.1016/j.marpolbul.2016.03.042 27038881 10.1016/j.marpolbul.2016.03.042

[CR9] Aghadadashi, V., Neyestani, M. R., Mehdinia, A., Riyahi Bakhtiari, A., Molaei, S., Farhangi, M., Esmaili, M., Rezai Marnani, H., & Gerivani, H. (2019). Spatial distribution and vertical profile of heavy metals in marine sediments around Iran’s special economic energy zone; Arsenic as an enriched contaminant. *Marine Pollution Bulletin,**138*, 437–450. 10.1016/j.marpolbul.2018.11.033 30660293 10.1016/j.marpolbul.2018.11.033

[CR10] Ahmed, M., & Abdel-Moati, M. (2003). Metal accumulation in sediments of the exclusive economic zone of Qatar, Persian Gulf. *International Journal of Environmental Sciences,**60*(1), 45–54. 10.1080/00207230304745

[CR11] Al-Arfaj, A. A., & Alam, I. A. (1993). Chemical characterization of sediments from the Gulf area after the 1991 oil spill. *Marine Pollution Bulletin,**27*, 97–101. 10.1016/0025-326X(93)90013-A

[CR12] Alharbi, T., Alfaifi, H., & El-Sorogy, A. (2017). Metal pollution in Al-Khobar seawater, Arabian Gulf, Saudi Arabia. *Marine Pollution Bulletin,**119*(1), 407–415. 10.1016/j.marpolbul.2017.03.011 28343632 10.1016/j.marpolbul.2017.03.011

[CR13] Al-Husaini, I., Abdul-Wahab, S., Ahamad, R., & Chan, K. (2014). Levels of Cd, Cu, Pb, and V in marine sediments in the vicinity of the Single Buoy Moorings (SBM3) at Mina Al Fahal in the Sultanate of Oman. *Marine Pollution Bulletin,**83*(1), 337–342. 10.1016/j.marpolbul.2014.04.006 24775070 10.1016/j.marpolbul.2014.04.006

[CR14] Ali, A., & Chidambaram, S. (2021). Assessment of trace inorganic contaminants in water and sediment to address its impact on common fish varieties along Kuwait Bay. *Environmental Geochemistry and Health,**43*(2), 855–883. 10.1007/s10653-020-00559-6 32335845 10.1007/s10653-020-00559-6

[CR15] Al-Jaberi, M. H., & Al-Dabbas, M. A. (2014). Assessment of heavy metals pollution in the sediments of Iraqi coastlines. *Science Journal,**3*(9), 2277–8179.

[CR16] Al-Kahtany, K., & El-Sorogy, A. S. (2023). Contamination and health risk assessment of surface sediments along Ras Abu Ali Island, Saudi Arabia. *Journal of King Saud University,**35*(2), 102509. 10.1016/j.jksus.2022.102509

[CR17] Al-Kahtany, K., El-Sorogy, A., Al-Kahtany, F., & Youssef, M. (2018). Heavy metals in mangrove sediments of the central Arabian Gulf shoreline, Saudi Arabia. *Arabian Journal of Geosciences,**11*, 1–12. 10.1007/s12517-018-3463-0

[CR18] Al-Kahtany, K., Nour, H. E., El-Sorogy, A. S., & Alharbi, T. (2023). Ecological and health risk assessment of heavy metals contamination in mangrove sediments, Red Sea coast. *Marine Pollution Bulletin,**192*, Article 115000. 10.1016/j.marpolbul.2023.115000 37210984 10.1016/j.marpolbul.2023.115000

[CR19] Al-Kahtany, K., Nour, H. E., Giacobbe, S., Alharbi, T., & El-Sorogy, A. S. (2023). Heavy metal pollution in surface sediments and human health assessment in southern Al-Khobar coast, Saudi Arabia. *Marine Pollution Bulletin,**187*, 114508. 10.1016/j.marpolbul.2022.114508 36603236 10.1016/j.marpolbul.2022.114508

[CR20] Allami, H., Afzali, A., & Mirzaei, R. (2020). Determination and investigation of heavy metal concentrations in sediments of the Persian Gulf coasts and evaluation of their potential environmental risk. *Analytical Methods in Environmental Chemistry Journal,**3*(4), 60–71. 10.24200/amecj.v3.i04.122

[CR21] Allen, H. E. (1995). *Metal Contaminated Aquatic Sediments* (p. 14). Ann Arbor Press.

[CR22] Al-Muzaini, S. M., & Jacob, P. G. (1996). An assessment of toxic metals content in the marine sediments of the Shuaiba Industrial Area, Kuwait, after the oil spill during the Gulf War. *Water Science and Technology,**34*, 203–210. 10.1016/S0273-1223(96)00746-9

[CR23] Al-Naimi, H. A., Al-Ghouti, M. A., Al-Shaikh, I., Al-Yafe, M., & Al-Meer, S. (2015). Metal distribution in marine sediment along the Doha Bay, Qatar. *Environmental Monitoring and Assessment,**187*, 1–14. 10.1007/s10661-015-4352-6 25701472 10.1007/s10661-015-4352-6

[CR24] Alomary, A. A., & Belhadj, S. (2007). Determination of heavy metals (Cd, Cr, Cu, Fe, Ni, Pb, Zn) by ICP-OES and their speciation in Algerian Mediterranean Sea sediments after a five-stage sequential extraction procedure. *Environmental Monitoring and Assessment,**135*, 265–280. 10.1007/s10661-007-9648-8 17342430 10.1007/s10661-007-9648-8

[CR25] Al-Qattan, F., & Al-Sarawi, M. (2017). Water quality assessment and bottom sediment in the coastal area of the three refineries (Mina Al-Ahmadi, Mina Shuaiba, and Mina Abdullah) Kuwait. *Journal of Environmental Pollution and Human Health,**5*, 69–82.

[CR26] Al-Rashdi, S., Arabi, A. A., Howari, F. M., & Siad, A. (2015). Distribution of heavy metals in the coastal area of Abu Dhabi in the United Arab Emirates. *Marine Pollution Bulletin,**97*(1–2), 494–498. 10.1016/j.marpolbul.2015.05.052 26081249 10.1016/j.marpolbul.2015.05.052

[CR27] Al-Saad, H. T., Abd, I. A., Al-Hello, M. A., & Zukhair, M. K. (2007). Environmental assessment of trace metals pollution in sediment of Khor al-Zubair Iraq. *Marina Mesopotamica,**21*(2), 23–33.

[CR28] Alshahri, F. (2017). Heavy metal contamination in sand and sediments near disposal site of reject brine from desalination plant, Arabian Gulf: Assessment of environmental pollution. *Environmental Science and Pollution Research,**24*(2), 1821–1831. 10.1007/s11356-016-7961-x 27796991 10.1007/s11356-016-7961-x

[CR29] Alshemmari, H., & Talebi, L. (2019). Heavy metal concentrations in the surface sediments of the northwestern Arabian Gulf Kuwait. *Arabian Journal of Geosciences,**12*, 1–9. 10.1007/s12517-019-4751-z

[CR30] Alshuiael, S., Alsaadi, H., & Hassan, H. (2022). Trace metal speciation within sediment from the Arabian Gulf. *Regional Studies in Marine Science,**56*, Article 102706. 10.1016/j.rsma.2022.102706

[CR31] Al-Taani, A. A., Batayneh, A., Nazzal, Y., Ghrefat, H., Elawadi, E., & Zaman, H. (2014). Status of trace metals in surface seawater of the Gulf of Aqaba, Saudi Arabia. *Marine Pollution Bulletin,**86*, 582–590. 10.1016/j.marpolbul.2014.05.060 25110052 10.1016/j.marpolbul.2014.05.060

[CR32] Alzahrani, H., El-Sorogy, A. S., Qaysi, S., & Alshehri, F. (2023). Contamination and risk assessment of potentially toxic elements in coastal sediments of the area between Al-Jubail and Al-Khafji, Arabian Gulf, Saudi Arabia. *Water (Basel),**15*(3), Article 573. 10.3390/w15030573

[CR33] Amin, S. A., & Almahasheer, H. (2022). Pollution indices of heavy metals in the Western Arabian Gulf coastal area Egyptian. *Journal of Aquatic Research,**48*(1), 21–27. 10.1016/j.ejar.2021.10.002

[CR34] Amin, B., Ismail, A., Arshad, A., Yap, C. K., & Kamarudin, M. S. (2009). Anthropogenic impacts on heavy metal concentrations in the coastal sediments of Dumai, Indonesia. *Environmental Monitoring and Assessment,**148*(1–4), 291–305. 10.1007/s10661-008-0159-z 18274874 10.1007/s10661-008-0159-z

[CR35] Arfaeinia, H., Dobaradaran, S., Moradi, M., Pasalari, H., Mehrizi, E. A., Taghizadeh, F., Esmaili, A., & Ansarizadeh, M. (2019). The effect of land use configurations on concentration, spatial distribution, and ecological risk of heavy metals in coastal sediments of the northern part along the Persian Gulf. *Science of the Total Environment,**653*, 783–791. 10.1016/j.scitotenv.2018.11.009 30759604 10.1016/j.scitotenv.2018.11.009

[CR36] Basaham, A. S., & El-Sayed, M. A. (1998). Distribution and phase association of some major and trace elements in the Arabian Gulf sediments Estuarine. *Coastal and Shelf Science,**46*(2), 185–194. 10.1006/ecss.1997.0278

[CR37] Bersuder, P., Smith, A. J., Hynes, C., Warford, L., Barber, J. L., Losada, S., Limpenny, C., Khamis, A. S., Abdulla, K. H., Le Quesne, W. J. F., & Lyons, B. P. (2020). Baseline survey of marine sediments collected from the Kingdom of Bahrain: PAHs, PCBs, organochlorine pesticides, perfluoroalkyl substances, dioxins, brominated flame retardants, and metal contamination. *Marine Pollution Bulletin,**161*, Article 111734. 10.1016/j.marpolbul.2020.111734 33065395 10.1016/j.marpolbul.2020.111734

[CR38] Castillo, A. B., El-Azhary, M., Sorino, C., & LeVay, L. (2024). Potential ecological risk assessment of microplastics in coastal sediments: Their metal accumulation and interaction with sedimentary metal concentration. *Science of the Total Environment,**906*, Article 167473. 10.1016/j.scitotenv.2023.167473 37778558 10.1016/j.scitotenv.2023.167473

[CR39] Chakraborty, P., Raghunadh Babu, P. V., Acharyya, T., & Bandyopadhyay, D. (2010). Stress and toxicity of biologically important transition metals (Co, Ni, Cu, and Zn) on phytoplankton in a tropical freshwater system: An investigation with pigment analysis by HPLC. *Chemosphere,**80*, 548–553. 10.1016/j.chemosphere.2010.04.039 20493512 10.1016/j.chemosphere.2010.04.039

[CR40] Chakraborty, P., Ramteke, D., Chakraborty, S., & Nath, B. N. (2014). Changes in metal contamination levels in estuarine sediments around India–an assessment. *Marine Pollution Bulletin,**78*(1–2), 15–25. 10.1016/j.marpolbul.2013.09.044 24211100 10.1016/j.marpolbul.2013.09.044

[CR41] Cuong, D. T., & Obbard, J. P. (2006). Metal speciation in coastal marine sediments from Singapore using a modified BCR-sequential extraction procedure. *Applied Geochemistry,**21*, 1335–1346. 10.1016/j.apgeochem.2006.05.001

[CR42] De Mora, S., Fowler, S. W., Wyse, E., & Azemard, S. (2004). Distribution of heavy metals in marine bivalves, fish, and coastal sediments in the Gulf and Gulf of Oman. *Marine Pollution Bulletin,**49*(5–6), 410–424. 10.1016/j.marpolbul.2004.02.029 15325209 10.1016/j.marpolbul.2004.02.029

[CR43] Egbueri, J.C., Ayejoto, D.A., Agbasi, J.C., 2022. Pollution assessment and estimation of the percentages of toxic elements to be removed to make polluted drinking water safe: A case from Nigeria. Toxin Rev. 1–15. 10.1080/15569543.2021.2025401

[CR44] El-Sorogy, A. S., Tawfik, M., Almadani, S. A., & Attiah, A. (2016). Assessment of toxic metals in coastal sediments of the Rosetta area, Mediterranean Sea, Egypt. *Environmental Earth Sciences,**75*, Article 398.

[CR45] El-Sorogy, A. S., Alharbi, T., & Richiano, S. (2018). Bioerosion structures in high-salinity marine environments: A case study from the Al–Khafji coastline Saudi Arabia. *Estuar Coast Shelf Science,**204*, 264–272. 10.1016/j.ecss.2018.03.005

[CR46] El-Sorogy, A. S., Al-Kahtany, K. (2022). Contamination and health risk assessment of heavy metals in surface sediments from the Arabian Gulf, Saudi Arabia. 10.21203/rs.3.rs-1586109/v1

[CR47] El-Tokhi, M., Amin, B. M., & Alaabed, S. A. (2017). Environmental assessment of heavy metals contamination of bottom sediments of Oman Gulf, United Arab Emirates. *Journal Pollution Effect Control,**5*, 203. 10.4172/2375-4397.1000203

[CR48] Fowler, S. W., Readman, J. W., Oregioni, B. J., Villeneuve, J. P., & McKay, K. (1993). Petroleum hydrocarbons and trace metals in nearshore Gulf sediments and biota before and after the 1991 war: An assessment of temporal and spatial trends. *Marine Pollution Bulletin,**27*, 171–182. 10.1016/0025-326X(93)90022-C

[CR49] Ghaemi, M., Soleimani, F., & Gholamipour, S. (2023). Heavy metal and persistent organic pollutant profile of sediments from marine protected areas: The northern Persian Gulf. *Environmental Science and Pollution Research,**30*(57), 120877–120891. 10.1007/s11356-023-30688-1 37945966 10.1007/s11356-023-30688-1

[CR50] Hakanson, L. (1980). An ecological risk index for aquatic pollution control, a sedimentological approach. *Water Research,**14*, 975–1001. 10.1016/0043-1354(80)90143-8

[CR51] Hamzeh, M. A., Shah-Hosseini, M., & Naderi Beni, A. (2013). Effect of fishing vessels on trace metal contamination in sediments of three harbors along the Iranian Oman Sea coast. *Environmental Monitoring and Assessment,**185*(2), 1791–1807. 10.1007/s10661-012-2668-z 22644121 10.1007/s10661-012-2668-z

[CR52] Hasna, V. M., Aboobacker, V. M., Dib, S., Izza, A., Yigiterhan, O., Al-Ansari, E. M., & Vethamony, P. (2024). Elemental distributions in the marine sediments off Doha, Qatar: Role of urbanization and coastal dynamics. *Environmental Earth Sciences,**83*(14), 434. 10.1007/s12665-024-11738-4

[CR53] Hassan, H., Benvenuto, C., Al-Maslamani, I., Chatting, M., Mondal, D., & Leitão, A. (2022). Methylmercury, trace metals, organotins, and their effects on the Qatari mangrove shrimp, Palaemon khori. *Journal of Marine Science and Engineering,**10*(7), Article 843. 10.3390/jmse10070843

[CR54] Houbolt, J. J. H. C. (1957). Surface sediments of the Persian Gulf near the Qatar Peninsula (p. 113). PhD Thesis, University of Albright, Des Haal Montana Co.

[CR55] Irandoost, F., Agah, H., Rossi, L., Calizza, E., Careddu, G., & Costantini, M. L. (2021). Stable isotope ratios (δ13C and δ15N) and heavy metal levels in macroalgae, sediment, and benthos from the northern parts of Persian Gulf and the Gulf of Oman. *Marine Pollution Bulletin,**163*, 111909. 10.1016/j.marpolbul.2020.111909 33486406 10.1016/j.marpolbul.2020.111909

[CR56] Janadeleh, H., & Jahangiri, S. (2016). Risk assessment and heavy metal contamination in fish (Otolithes ruber) and sediments in the Persian Gulf. *Journal Community Health Research,**5*(3), 169–181.

[CR57] Janadeleh, H., & Kameli, M. A. (2017). Metals contamination in sediment and their bioaccumulation in plants and three fish species from freshwater ecosystem. *Toxins Reviews,**36*(4), 297–305. 10.1080/15569543.2017.1309551

[CR58] Janadeleh, H., Jahangiri, S., & Kameli, M. A. (2018). Assessment of heavy metal pollution and ecological risk in marine sediments (a case study: Persian Gulf). *Human and Ecological Risk Assessment,**24*(8), 2265–2274. 10.1080/10807039.2018.1443792

[CR59] Juma, H. (2005). Trace metals in marine sediment of Bahrain territorial water (p. 26). Report to Public Commission for the Protection of Marine Resources, Environment, and Wildlife.

[CR60] Karbassi, A., & Bayati, A. (2005). Environmental geochemistry of heavy metals in a sediment core off Bushehr, Persian Gulf. *Journal of Environmental Health Science and Engineering,**2*, 255–260.

[CR61] Loughland, R.A., AlAbdulkader, K.A., Wyllie, A., Burwell, B.O., 2012. Anthropogenic induced geomorphological change along the Western Arabian Gulf coast. In: Piacentini, T. (Ed.), Studies on Environmental and Applied Geomorphology. InTech, 191–218. 10.5772/28330

[CR62] Lyons, B. P., Barber, J. L., Rumney, H. S., Bolam, T. P. C., Bersuder, P., Law, R. J., & Al-Sarawi, H. A. (2015). Baseline survey of marine sediments collected from the State of Kuwait: PAHs, PCBs, brominated flame retardants and metal contamination. *Marine Pollution Bulletin,**100*(2), 629–636. 10.1016/j.marpolbul.2015.08.014 26344820 10.1016/j.marpolbul.2015.08.014

[CR63] Mahfouz, M. M., Yigiterhan, O., Elnaiem, A. E., Hassan, H. M., & Alfoldy, B. (2019). Elemental compositions of particulate matter retained on air condition unit’s filters at Greater Doha, Qatar. *Environmental Geochemistry and Health,**41*, 2533–2548. 10.1007/s10653-019-00304-8 31054073 10.1007/s10653-019-00304-8 PMC6856027

[CR64] Massoud, M. S., Al-Abdali, F., Al-Ghadban, A. N., & Al-Sarawi, M. (1996). Bottom sediments of the Arabian Gulf—II. TPH and TOC contents as indicators of oil pollution and implications for the effect and fate of the Kuwait oil slick. *Environmental Pollution,**93*(3), 271–284. 10.1016/S0269-7491(96)00042-5 15093526 10.1016/s0269-7491(96)00042-5

[CR65] Mirzaei, M., Hatamimanesh, M., Haghshenas, A., Moghaddam, S. M., Ozunu, A., & Azadi, H. (2020). Spatial-seasonal variations and ecological risk of heavy metals in Persian Gulf coastal region: Case study of Iran. *Journal of Environmental Health Science and Engineering,**18*(1), 91–105. 10.1007/s40201-019-00441-3 32399223 10.1007/s40201-019-00441-3 PMC7203289

[CR66] Mukhopadhyay, A., Al-Sulaimi, J., Al-Awadi, E., & Al-Ruwaih, F. (1996). An overview of the Tertiary geology and hydrogeology of the northern part of the Arabian Gulf region with special reference to Kuwait. *Earth-Science Reviews,**40*(3–4), 259–295. 10.1016/0012-8252(95)00068-2

[CR67] Muller, G. (1969). Index of geoaccumulation in sediments of the Rhine River. *Geo Journal,**2*, 108–118.

[CR68] Naser, H. A. (2013). Assessment and management of heavy metal pollution in the marine environment of the Arabian Gulf: A review. *Marine Pollution Bulletin,**72*(1), 6–13. 10.1016/j.marpolbul.2013.04.030 23711845 10.1016/j.marpolbul.2013.04.030

[CR69] Nour, H. E., & Nouh, E. (2020). Using coral skeletons for monitoring of heavy metals pollution in the Red Sea Coast. *Egypt. Arab J. Geosci.,**13*, 340. 10.1007/s12517-020-05308-8

[CR70] Nour, H. E., Alshehri, F., Sahour, H., & El-Sorogy, A. S. (2022). Evaluation of sediment and water quality of Ismailia Canal for heavy metal contamination, Eastern Nile Delta, Egypt. *Regional Studies in Marine Science,**56*, Article 102714. 10.1016/j.rsma.2022.102714

[CR71] Nour, H. E., Helal, S. A., & Wahab, M. A. (2022). Contamination and health risk assessment of heavy metals in beach sediments of Red Sea and Gulf of Aqaba, Egypt. *Marine Pollution Bulletin,**177*, Article 113517. 10.1016/j.marpolbul.2022.113517 35299149 10.1016/j.marpolbul.2022.113517

[CR72] Peer, F. E., & Safahieh, A. (2011). Temporal and spatial variations of heavy metals (Cu, Pb, and Ni) concentration in the sediments from intertidal zone along the Iranian coasts of the Oman Sea. *J. Oceanogr. Mar. Sci.,**2*(7), 158–164.

[CR73] Pourang, N., Nikouyan, A., & Dennis, J. H. (2005). Trace element concentrations in fish, surficial sediments and water from northern part of the Persian Gulf. *Environmental Monitoring and Assessment,**109*, 293–316. 10.1007/s10661-005-6287-9 16240204 10.1007/s10661-005-6287-9

[CR74] Purser, B.H., Seibold, E., 1973. The principal environmental factors influencing Holocene sedimentation and diagenesis. In: Purser, B.H. (Ed.), The Persian Gulf—Holocene Carbonate Sedimentation and Diagenesis in a Shallow Epicontinental Sea. Springer, New York, 1–9. 10.1007/978-3-642-65545-6_1

[CR75] Reynolds, R. M. (1993). Physical oceanography of the Gulf, Strait of Hormuz, and the Gulf of Oman: Results from the Mt. Mitchell expedition. *Marine Pollution Bulletin,**27*, 35–59. 10.1016/0025-326X(93)90007-7

[CR76] Ruilian, Y. U., Xing, Y., Yuanhui, Z., Gongren, H. U., & Xianglin, T. U. (2008). Heavy metal pollution in intertidal sediments from Quanzhou Bay China. *Journal Environment Science,**20*, 664–669. 10.1016/s1001-0742(08)62110-5 10.1016/s1001-0742(08)62110-5 18763559

[CR77] Ruiź-Fernández, A. C., Hillaire-Marcel, C., Paez-Osuna, F., Ghaleb, B., & Soto Jimenez, M. (2003). Historical trends of metal pollution recorded in sediments of the Culiacan River Estuary, Northwestern Mexico. *Applied Geochemistry,**18*, 577–588. 10.1016/S0883-2927(02)00117-8

[CR78] Sheppard, C., Al-Husiani, M., Al-Jamali, F., Al-Yamani, F., Baldwin, R., Bishop, J., Benzoni, F., Dutrieux, E., Dulvy, N. K., Durvasula, S. R. V., Jones, D. A., Loughland, R., Medio, D., Nithyanandan, M., Pilling, G. M., Polikarpov, I., Price, A. R. G., Purkis, S., Riegl, B., … Zainal, K. (2010). The gulf: A young sea in decline. *Marine Pollution Bulletin,**60*(1), 13–38. 10.1016/j.marpolbul.2009.10.017 20005533 10.1016/j.marpolbul.2009.10.017

[CR79] Shriadah, M. A. (1998). Metals pollution in marine sediments of the United Arab Emirates creeks along the Arabian Gulf shoreline. *Bulletin of Environmental Contamination and Toxicology,**60*, 417–424. 10.1007/s001289900642 9528701 10.1007/s001289900642

[CR80] Smrzka, D., Zwicker, J., Bach, W., Feng, D., Himmler, T., Chen, D., & Peckmann, J. (2019). The behavior of trace elements in seawater, sedimentary pore water, and their incorporation into carbonate minerals: A review. *Facies,**65*, 1–47. 10.1007/s10347-019-0581-4

[CR81] Soylak, M., Divrikli, U., Saracoglu, S., & Elci, L. (2002). Monitoring trace metal levels in Yozgat-Turkey: Copper, iron, nickel, cobalt, lead, cadmium, manganese, and chromium levels in stream sediments. *Polish Journal of Environmental Studies,**11*(1), 47–51.

[CR82] Taylor, S. R., & McLennan, S. M. (1995). The geochemical evolution of the continental crust. *Reviews of Geophysics,**33*, 241–265. 10.1029/95RG00262

[CR83] Tomlinson, D. L., Wilson, J. G., Harris, C. R., & Jeffrey, D. W. (1980). Problems in the assessment of heavy-metal levels in estuaries and the formation of a pollution index. *Helgoland Meeresuntersuchungen,**33*, 566–575. 10.1007/BF02414780

[CR84] Veerasingam, S., Venkatachalapathy, R., & Ramkumar, T. (2012). Heavy metals and ecological risk assessment in marine sediments of Chennai India. *Carpathian Journal Earth Environment Science,**7*, 111–124.

[CR85] Veerasingam, S., Venkatachalapathy, R., & Ramkumar, T. (2014). Historical environmental pollution trend and ecological risk assessment of trace metals in marine sediments off Adyar estuary, Bay of Bengal, India. *Environmental Earth Sciences,**71*, 3963–3975. 10.1007/s12665-013-2781-5

[CR86] Veerasingam, S., Vethamony, P., Mani Murali, R., & Fernandes, B. (2015). Depositional record of trace metals and degree of contamination in core sediments from the Mandovi estuarine mangrove ecosystem, west coast of India. *Marine Pollution Bulletin,**91*, 362–367. 10.1016/j.marpolbul.2014.11.045 25510546 10.1016/j.marpolbul.2014.11.045

[CR87] Venkatachalapathy, R., Veerasingam, S., Basavaiah, N., Ramkumar, T., & Deenadayalan, K. (2011). Environmental magnetic and geochemical characteristics of Chennai coastal sediments, Bay of Bengal India. *Journal Earth System Science,**120*, 885–895. 10.1016/j.marpolbul.2011.01.030

[CR88] Yigiterhan, O., Al-Ansari, E. M., Nelson, A., Abdel-Moati, M. A., Turner, J., Alsaadi, H. A., Paul, B., Al-Maslamani, I. A., Al-Yafei, M. A., & Murray, J. W. (2020). Trace element composition of size-fractionated suspended particulate matter samples from the Qatari Exclusive Economic Zone of the Arabian Gulf: The role of atmospheric dust. *Biogeosciences,**17*(2), 381–404. 10.5194/bg-17-381-2020

[CR89] Zheng, N. A., Wang, Q., Liang, Z., & Zheng, D. (2008). Characterization of heavy metal concentrations in the sediments of three freshwater rivers in Huludao City Northeast China. *Environment Pollution,**154*(1), 135–142. 10.1016/j.envpol.2008.01.001 10.1016/j.envpol.2008.01.001 18280624

