# Supplementary material for: Long-term trends in heavy metal contamination of marine sediments in the Arabian Gulf: A meta-analysis
Source: Environ Monit Assess. 2025 Jul 8;197(8):873. doi: 10.1007/s10661-025-14348-0 (PMC12238209; doi:10.1007/s10661-025-14348-0)
Supplement: Supplementary file 1 — Supplementary file1 (DOCX 42 KB) [file 10661_2025_14348_MOESM1_ESM.docx]

| **Table S1** Grade for ERI | | | | | | |
| --- | --- | --- | --- | --- | --- | --- |
| **Risk level** | **Low risk** | **Moderate risk** | **High risk** | **Very high risk** | **Disastrous risk** |  |
| Grade | I | II | III | IV | V |  |
| ERI | < 30 | 30–60 | 60–120 | 120–240 | > 240 |  |

**Table S3** Environmental and Methodological Factors Influencing HM Variability

| SL NO: | Sample type | Season | Grain size | Minerology | Analytical methods used | Reference |
| --- | --- | --- | --- | --- | --- | --- |
| 1 | Near shore Surface sediments (0-2 cm) | June to October; Summer and early autumn months | Not mentioned | approximately 400 km of the oil spill release sites.  Not mentioned | micro- wave digestion, then taken to dryness and the residue  taken up in 2% nitric acid for analysis by ICP-MS. | Fowler et al. (1993) |
| 2 | Sediment samples were collected from the Abu Ali area of  inter-tidal zone and in benthic locations. | Not mentioned | The samples were primarily clayish, except for TR-3 which is sandy and has some broken shell, at southeast side of Jinna the sample was clayish and sandy and TR-7 which was soft, sandy and clayish.7 | Abu Ali area  Not mentioned | 10 ml concentrated nitric acid and 3 ml concentrated perchloric acid. The mixture was digested at 120°C for 3 h  Inductively Coupled  Argon Plasma (ICAP) analyser. | AL-Arafaj & Alam (1993) |
| 3 | Sediment samples were collected from the top 4-5 cm of the bottom sediments  The study focused on marine sediments in the Shuaiba Industrial Area | Samples were collected between August 1993 and June 1994  The collection period spanned summer and spring seasons | Sediment samples were sieved with a 63 µm mesh sieve  Most samples were clayish, except sandy samples from station  The study involved marine sediments from the Shuaiba Industrial Area | Shuaiba Industrial Area (SIA)  Not mentioned | Sediment samples were acid digested in a block digester  Analyses were performed using a Shimadzu model AA-680 atomic absorption spectrophotometer | AI-Muzaini & Jacob (1996) |
| 4 | Surface sediments were collected from 24 stations in the Gulf  Bottom sediments were analyzed for elemental composition and grain size  The study focused on sediment types from the Arabian Gulf | Not mentioned | Grain size analysis showed muddy sediments dominate the northern Gulf  The southern Gulf is characterized by sand-sized sediments  Sediments are arranged in bands parallel to the Gulf's main axis  Average grain size increases from north to south | Two major sediment types identified: terrigenous fine-grained and Al rich type predominating along the Iranian side and a coarse-grained and carbonate rich type predominating along the Arabian side of the Gulf. | Trace metals were analyzed using inductively coupled plasma atomic emission spectrophotometry (ICP/AES)  Atomic absorption spectrophotometry (AAS) was used for measuring Mn, Zn, Cu, and Ni | Basaham and El-Sayed (1998) |
| 5 | The study involved surficial sediment samples from various stations  Samples were collected from the near-shore area, indicating surface sediments  Mud and sand sediments were texturally classified, representing bottom sediments | Not mentioned | Sediments consist of sand and mud textural classes    Kuwait area sediments are primarily mud-sized  Sediments transition to sand-size materials south of Kuwait | The elements analysed were high in the mud  samples from the Kuwait area and decrease to the south  with an increasing sand fraction to attain their lowest  concentration in the deposits of **Qata**r.  ain their lowest concentration in the deposits of Qatar. The increase of trace element contents in the sand sediments of Saudi Arabia could be due to the presence of detrital sand- size particles rich in trace elements. | Trace metal analysis was performed using Inductively Coupled Plasma Atomic Emission Spectroscopy (ICP/AES) | Basaham & El-Sayed (1993) |
| **6** | **Off Shore Surface Sediment** samples were collected from creeks along the Arabian Gulf Coast depths of 16 to 18 m |  | Sediment samples were collected using a Hydro-Bios grap sampler  Fractions with particles larger than 500 m, 500–250 m,  250–125 m, 125–63 m, and less than 63 m (Table I) were taken for further  analysis. | Sediment  Muddy type | Trace metals were analyzed using atomic absorption spectrophotometry  Samples were treated with hydrogen peroxide and hydrochloric acid  Measurements were performed in duplicate for accuracy | Shriadah et al. (1998) |
| 7 | Surface sediments were collected directly into pre-cleaned Teflon containers  Sediment samples were collected from coastal locations during 2000-2001  The study assessed marine contamination based on coastal sediments | Bimonthly sampling  Spring (March and April)  Summer (July and August)  Autumn (Nov) | Sediment samples were sieved through a 1 mm sieve  Smaller samples were ground in an agate mortar  Samples were transferred to zip-lock bags for homogenization | The geology of Oman and eastern UAE is rich in ophiolites  Ophiolites contain chromite and nickel sulfide minerals  Coastal sediments reflect local mineralogy due to low industrial activity | Sediments were analyzed using cold vapour atomic fluorescence spectrometry (CV-AFS)  Cold vapour atomic absorption spectrometry (CV-AAS) was also employed  ICP-MS was used for analyzing sediments from Oman | De Mora et al. (2004) |
| 8 | Twenty-three bottom sediment samples were collected from the EEZ of Qatar  Samples were analyzed to determine seabed structure and chemical composition  Bottom sediments are the focus for pollutant concentration analysis | Not mentioned | Mean grain size ranged from 1.34 mm to 3.86 mm.  Average grain size was 2.50 mm.  Gulf mean grain size varied between 0.3 and 6.74 mm.  Coarse sand had elevated carbonate content exceeding 95% | Carbonate sediments, Sand texture size sediments are composed of ooids, calcareous shells and shell fragments of calcareous organisms. | Heavy metals were analyzed using Atomic Absorption Spectrophotometer (AAS) Model 2380  Metals concentrations were analyzed in triplicates for precision and accuracy  The CRM-1645 was used to check accuracy | Ahmed, M.& Abdel-Moati, M. (2003) |
| 9 | Surface sediments | Not mentioned | 1 mm sieve | Not mentioned | Not mentioned | Juma et al. (2005) |
| 10 | The study collected surface sediment samples from the Persian Gulf  Sediment samples were analyzed for trace metals and major elements  Samples were collected from 27 stations in 9 transects | Sediment samples were collected in January 2004 | Sediment samples were ground to achieve fine homogenized sediment  The sediment was passed through a 1mm PE sieve  Fine-grained sediments acted as good absorbents for pollutants | Soft sediments | Inductively Coupled Plasma Mass Spectrometry (ICP MS) was used for trace metals | Agah et al. (2012) |
| 11 | Surficial sediments were collected from all sampling sites  Sediment samples were passed through a 62-micron sieve  Bottom sediments were analyzed for trace metal concentrations | Samples were collected during summer and winter seasons  Sampling occurred in February (winter) and July (summer) 2003 | Sediment grain size affects trace metal concentrations significantly  Fine grain fractions (<63 µm) were analyzed in sediments  Coarser particles may indicate anthropogenic inputs due to limited transport  Sampling sites showed variations in fine grain proportions |  | Inductively Coupled Plasma Mass Spectrometer was used for analysis  Analyses were conducted in triplicate for accuracy  Certified reference materials were analyzed to assess accuracy | Pourang et al. (2005) |
| 12 | Surficial sediment samples | summer 2006 | <63 um fraction of the sediment |  | Atomic Absorption Spectrometric analysis. | Al Saad et al., 2007 |
| 13 | Sediment samples were taken from 3 to 5 cm surface  The study focused on surface sediments in the intertidal zone | Samples were collected in August 2007 and February 2008  August represents summer, while February represents winter | Samples were sieved with a 63 µm stainless steel sieve  Sediment samples were taken from 3 to 5 cm surface  No specific grain size measurements were provided in the paper | Not mentioned | Heavy metals were analyzed using atomic absorption spectrophotometry method  Samples were acid digested before analysis  Concentrations were expressed as micrograms per gram in dry weight | Peer & Safahieh (2011) |
| 14 | Surface and bottom sediments were collected from nine stations | April 2001 | Mean grain size (Mz) was measured for sediment samples  Coarsest sand fractions were found at the shallowest station  Grain size distribution correlates with contaminant levels | Carbonate Minerals | Heavy metals were analyzed using ICP-AES method.  Samples were digested with HCl and HNO3. | Abd El-Gawad et al. (2008) |
| 15 | Sediment samples were collected from surface sediments | Samples were collected in January 2010 at low tide  The study does not specify other seasons for sample collection. | 63µm mesh sieve | Not mentioned | Metals aluminum (AL), iron (Fe), lead (Pb), manganese (Mn), nickel (Ni), vanadium (V), zinc (Zn) and copper (Cu) were determined by ‘‘Inductively Coupled Plasma Atomic Emission Spectrophotometery’’  Mercury concentrations were determined using a Zeeman Mercury Analyzer RA-915+ | Abdollahi et al. (2013) |
| 16 | Surface sediments (0-5 cm)  A core sample was also analyzed alongside surface sediments | Not mentioned | The mean size 88.68 µm of deposited particles is positively associated with carbonate levels  Finer deposited particles increase TOM levels significantly  The study indicates a dominance of sand fraction in sediments | Not mentioned | Trace elements were determined using ICP-OES method  Samples underwent acid digestion with Aqua regia  Bioavailable parts of As were measured using a graphite furnace system  Quality assurance utilized certified reference marine sediment IAEA-405  Working standards were prepared with serial dilution in ultrapure HNO3 | Aghadadashi et al. (2019) |
| 17 | Surface sediments | May and December 2012 | Not mentioned |  | ICP-OES. | Al-Naimi et al., 2015 |
| 18 | The study examined surface sediments in three harbors  A total of 28 bottom sediment samples were collected  Beach samples were collected as surface sediments | Samples were collected in February 2010 during the winter season  The winter season is characterized by the NE monsoon from November to March  The study reports seasonal variations affecting sediment characteristics | Most samples are very fine sands from Pasabandar harbors  Station P10 contains gravel and coarse-very coarse sands  Sediments are classified as clayey silt and sandy clayey silt  Outside harbors, sediments are mainly silty sand  63-μm screen used | The study area is part of the Makran accretionary wedge  It consists mainly of turbidites and ophiolitic mélanges  Sediments include calcareous mudstones and various minerals  Ophiolite mélange units contribute to metal contamination | ICP-AES | Hamzeh et al. (2013) |
| 19 | subtidal sediment samples | June 2009 to April 2010. | Grain size is influenced by trace metal concentration  Smaller grain sizes are associated with higher trace metal concentrations  Marine sediments' chemical composition depends on grain size  Sediment samples were sieved to analyze grain size distribution | Not mentioned | Aqua regia digestion method was used for analysis  Pseudo-total analysis was conducted using Vista ICP-OES | Al-Husaini et al. (2014) |
| 20 | Sediment samples were collected from the top layer of each grab sample  The study focused on marine sediment, indicating bottom sediment analysis | Not mentioned | Fine sediment fraction sample (<63μm) was used | Not mentioned | Sediment samples were digested using acids in a microwave system  Analysis was performed using ICP-MS and ICP-OES techniques  Trace elements were quantified by external calibration with indium as an internal standard | Lyons et al. (2015) |
| 21 | Surface sediments | Not mentioned |  |  | ICP-AES, ICP-MS | Al-Jaberi & Al-Dabbas, 2014 |
| 22 | Surface sediment s | Winter | Not mentioned |  | ICP technique for Hg, As and  Atomic Absorption Spectroscopy for Cd, Pb | Abdulnabi et al., 2019 |
| 23 | Surface coastal marine sediments were collected from eight sites | Samples were collected in the summer of 2015  Surface sediments were collected seasonally | Grain size fractions less than 63 mm were separated for analysis  Sediment samples were air-dried and sieved for grain size determination | Not mentioned | Sediment samples were analyzed using inductively coupled plasma-optical emission spectrometry (ICP-OES)  Samples were prepared according to MOOPAM guidelines  Sediments were digested using aqua regia before analysis | Janadeleh et al. (2018) |
| 24 | sediment samples from the Abu Dhabi coastal area | Not mentioned | Not mentioned | Not mentioned | Inductively Coupled Plasma-Mass Spectroscopy (ICP-MS) was used for analysis and  X-ray fluorescence was also employed for trace metal analysis | Al-Rashidi et al. (2015) |
| 25 | Surface sediments | April 2016 | Sediments included clays and very fine sands  2mm sieve used studied coast is distinguished into three types : 1) Sandy-dominated shores, composed of coarse sand, sandy mud and very few biogenicmaterials | Not mentioned | Inductively Coupled Plasma-Mass Spectrometer (ICP-MS) | Alharbi et al., (2017) |
| 26 | Surface sediments (0-5 cm) were collected using an Ekman grab sampler.  The study focused on sediment samples from the Persian Gulf and Gulf of Oman | May and June | Grain size was measured using laser-scattering and shaker instruments  Fine-size sediments (63 μ) were used for isotopic analysis  Sediments of size 150 μ were used for heavy metal analyses | The sediments were sandy with pebbles in structure  Northeastern and central Persian Gulf had muddy bed structures  Fine-size sediments were used for isotopic analysis  The study area includes northern Persian Gulf and Gulf of Oman | ICP-OES | Irandoost et al. (2021) |
| 27 | The study focused on surface sediments from Chabahar Bay | April | smaller than 63 μm  Thesand, siltandclaycontentsaveraged60±29.15%,29.48± 22.04%and10.51±10.81%respectively. | Sand% 60±29.15 Silt% 29.48±22.04 Clay% 10.51±10.81 | For digestion of sediment samples,0.5g of each sediment sample was added with a mixture of HCl–HNO3–H2O(with the ratio of 1:1:1v/v) andheatedat95°Cduring1h.Metal(Al,Co,Ni,Cu,Zn,As,  Pb,Fe,CrandV) analyses were performed using InductivelyCoupled  Plasma Mass Spectrometry inductively (ICP-MS) after acid digestion at ACME Lab.,Canadawhichisunder17,025 standards | Agah et al. (2016) |
| 28 | Surface sand samples were collected at 2 m from the shoreline  Sediment samples were taken from surface and 30 cm depth  Twelve sediments were collected from seawater during low tide | Not mentioned | Not mentioned | Not mentioned | Atomic absorption spectrometry (AAS) was used for trace metal analysis  [Energy dispersive X-ray fluorescence spectrometer (EDX) was also employed for Ca, Fe, Al, Ti, Sr, and Rb. | Alshahri et al. (2016) |
| 29 | Bottom Sediments | Samples were collected in spring, summer, autumn, and winter of 2017 | Not mentioned | Not mentioned | Sediment samples were dried at 100°C for 24 hours  One gram of each sample was digested with nitric and perchloric acids  The digested samples were filtered and diluted to 25 ml | Mirzaei et al. (2020) |
| 30 | The study analyzed surface sediments from Chabahar Bay.  Sediment samples were collected from various stations .  No specific mention of bottom sediments was found | July | Sediment samples consisted of sand, silt, and clay fractions.  Sand ranged from 11.7% to 100% in samples.  Silt levels varied from 0% to 85% .  Clay content was between 0% and 3.3% | The study area features oceanic crustal ophiolitic rocks  Geological nature includes natural sources of elements like Cr and Ni  Sediment samples consist of sand, silt, and clay | Inductively coupled plasma mass spectrometry (ICP-MS) was used for analysis | Agah, 2021 |
| 31 | Bottom sediment samples were collected from three refineries. | Not mentioned | Not mentioned | Not mentioned | Concentrations of trace metals were measured by the EPA 3050B method using instrument Perkin-Elmer-A Analyst 700. | Al-Qattan F., Al-Sarawi M. (2017) |
| 32 | The study collected bottom sediment samples from various locations |  | The mean grain size of 23 samples was studied  Khor Kalba samples are muddy sand to sand  Dibba area samples are sandy to gravelly sand  Khor Fakan samples have sand texture  Fujairah area samples range from muddy sand to sand | The study area features jagged mountains and oceanic rocks.  Bedrock consists of terrigenous sedimentary and shell fragments | Inductively Coupled Plasma-Mass Spectrometry (ICP-MS) was used for analysis  Samples were digested in hydrochloric and nitric acids  The leachate was filtered using Whitman No.1 filter pape | El-Tokhi et al. (2017) |
| 33 | Samples were collected from the highest intertidal level along the strandline.  Sediment samples were taken 3-4 cm from the surface | Samples were collected between May 2017 and May 2018 | Sediment particle size was measured using Malvern Mastersizer-3000 . The samples were dried at 50 ◦ C, homogenized and sieved through a 5 mm metallic sieve to separate the bulk sediments  Samples were classified by percentage sand, clay, and silt composition  Grain size analysis showed a sand percentage of 89-100% | The coastal area features quaternary deposits of carbonate rocks and sediments  Bedrock includes dolostones, indicating a carbonate geological nature  Tidal currents may influence sediment sorting and composition | Metals were extracted using HNO3 and HF in Teflon tubes  The solution was analyzed using ICP-OES after digestion. | Castillo et al. (2024) |
| 34 | Varying water depths | Not mentioned | Not mentioned | Not mentioned | Not mentioned | Alshuiael et al., 2022 |
| 35 | Surface sediments(1 to 30 cm deep) | Not mentioned | Not mentioned | littoral zone | ICP-MS | El-sorogy et al. (2018) |
| 36 | Eighteen surface sediment samples were collected from intertidal zone (1 to 40 cm depth) of natural mangroves.  Sediment samples were analyzed for heavy metals in surface layers | Not mentioned | Grain size analysis was performed on sediment samples.  Sediments comprised 55.75% sand, 43.45% silt, and 0.80% clay.  Samples were very fine, muddy, and silty.  Sediment samples were collected from 1 to 40 cm depth | Muddy sand | Trace metal analysis was performed using ICP-MS: NexION 300D | Al-Kahtany et al. (2018) |
| 37 | The study analyzed surface sediment samples | Not mentiioned | The mean silt content is 81.2% across samples  Sand fraction averages 12.6%, ranging from 0.91 to 39.7%  Clay fraction has a mean value of 6.2%  Grain size analysis involved 21 surface sediment samples | Autochthonous materials formed by the degradation of recent shells and the erosion of coastal sediments and the allochthonous materials derived from on  shore desert sediments, Dust storms composed of calcareous and quartz grains, which are mostly in the silt range. | Sediment samples underwent microwave digestion for analysis  Inductively coupled plasma mass spectrometry (ICP-MS) determined metal concentrations | Alshemmari et al. (2019) |
| 38 | Surface sediments, the top layer (1-2 cm) of each grab sample was analyzed | Samples were collected in May 2017 and November 2019  The study indicates two distinct sampling seasons | Particle size analysis was completed on each sediment samples | Not mentioned | Not mentioned | Bersuder et al. (2020) |
|  | Sediment samples were collected from coastal areas in Asalouyeh Town  The study focused on bottom sediments from various land use types | Samples were collected from November 2016 to January 2017.  This period corresponds to late autumn and early winter. | Sediment samples showed grain size distribution across various land use types  Dominant textures included sand and silt in all regions  Clay content ranged from 11.6% to 37.2%  Silt content varied between 23.43% and 61.32% |  | Heavy metals were analyzed using Atomic Absorption Spectrophotometer (AAS)  The method involved digestion with acids before analysis  Samples were filtered through polycarbonate filters prior to analysis | Arfaeinia et al. (2019) |
| 39 | surface and deep sediments samples | Not mentioned | 63-micron sieve | Not mentioned | Flame atomic absorption spectrometer (F-AAS), with an air-acetylene flame  GBC electrothermal atomic absorp tion spectrometer (ET-AAS, Plus 932, Australia) using a graphite furnace module (GF3000, GBC) was used for validation of results | Allami et al. (2020) |
| 40 | Surface sediment samples of offshore areas | Not mentioned | smallest granular portion was selected for analysis The dominant mesh size in sediments was silty particles | Grain size analysis indicated similar sedimentary texture in both regions | The measurement of the concentrations of heavy metals in samples was performed using inductively coupled plasma mass spec trophotometry (ICPMS). | Aali et al. (2024) |
| 41 | surface sediment samples | Not mentioned | Not mentioned | Sandy- -dominated shore, with rocky and mangrove in parts. | V, Fe, As, Co, Ni, Zn, Cr, Pb, and Cu were analysed using inductively coupled plasma-atomic emission spectrometry (ICP - AES) | El-Sorogy et al. (2022) |
| 42 | The study collected surface sediment samples | Winter | 63-μm sieve  Sand 4-94% and mud 0.13-95.08% studied |  | ICP-MS used | Ghaemi et al. (2023) |
| 43 | Sediment samples were collected from the bottom using Van Veen Grab  The study focused on marine sediments off Doha Bay | extremely hot summer (May-September) and a moderately cold winter (Novem ber-March) | The grain size distribution was analyzed using the Mastersizer 3000  Sediment at station S0 is mostly silty: 72% silt, 25% sand, 3% clay  Offshore stations are predominantly sandy, with 50-100% sand content | Due to the high particle size of sandy sediments, there is a lower accumulation of contami nants within the sediments  Moreover, low current speed and high residence time in the bay (Hanert et al. 2023) may allow the accumulation of metals in sediments, either from the sources of outfall or from dust deposition | Inductively Coupled Optical Emission-Mass Spectrometer (ICP-OES) analyzed trace elements | Hasna et al. (2024) |
| 44 | Top 5 cm of sediments | September to november | Sieved to remove particles>2mm. | Not mentioned | Inductively Coupled Plasma-Optical Emission Spectrometer-ICP-OES; Varian720-ES (Clescerietal.,1998). | Amin et al. (2022) |
| 45 | surface sediment samples | January | <63 lm  fraction for analysis. The samples of the lower HM levels, such as S10,S14,S16,S18,S19,andS21,are characterized by medium to coarse size and occurred in the north of the study area faced to the open sea. |  | ICP- AES | Al-Kahtany&El- Sorogy, 2023 |
| 46 | Surface sediment samples were collected from Ras Abu Ali Island  The study focused on coastal sediment contamination | Samples were collected in January 2021 | Samples were subjected to size fractionation using sieves for analysis.  The <63 µm fraction was obtained for analysis  Samples characterized by fine and very fine sized composition showed higher HM accumulation |  | Inductively coupled plasma-atomic emission spectrometry (ICP-AES) was used for analysis  Samples were digested with HNO3-HCl aqua regia before analysis | Alzahrani et al. (2023) |
| 47 | Sediment samples were collected from the intertidal zone at water depths of 0.15 to 0.50 m  The study focuses on coastal sediments between Al-Jubail and Al-Khafji  No specific mention of bottom or surface sediments is provided |  | Sediments were sieved into various size fractions: >500 μm to <63 μm  Dried sediments were ground to a particle size less than 150 μm  The study involved multiple size categories for sediment analysis | The study area is near Al-Jubail industrial city, rich in petrochemical activities  Coastal sediments contain biogenic and terrestrial components from hinterland Quaternary sediments  Lithogenic sources are indicated from weathering of rocks in the area  The geological nature includes sandy shorelines with rocky areas | Sediments were digested with aqua regia for analysis  Analysis performed using ICP-AES at ALS Geochemistry Lab, Jeddah  Detection limits for PTEs ranged from 1.0 to 10,000 mg/kg  Validation included precision checks and recovery values between 80-120% |  |
